# Competence and Care: A Scoping Review Exploring the Incorporation of Compassion and Relational Skills in Applied Behavior Analysis

**DOI:** 10.3390/bs16101950

**Published:** 2026-10-20

**Authors:** Daniele Nunes Longhi Aleixo, Danielle Lucila Ramos, Pedro Carlos Cagnazzo, Leonila Santos de Almeida Sasso, Amanda Bueno dos Santos, João Daniel de Souza Menezes, Matheus Querino da Silva, Emerson Roberto dos Santos, Gabriela dos Santos Longo Cesar, Ana Maria Rita Pedroso Vilela Torres de Carvalho Engel, Janaína Aparecida de Sales Floriano, Ana Paula Bernardes da Rosa, Adriana Viviane Vieira, Ana Beatriz Flandoli Valencio, Arthur de Castro Reis Mauricio, Clarissa Albuquerque Vaz Nunes, José Luis Esteves Francisco, Gustavo Gouveia Macedo, Andressa Karina Stefani, Camila Bortoluci de Lima, Maysa Alahmar Bianchin, Marli de Carvalho Jericó, Fabiana de Souza Orlandi, Luís Cesar Fava Spessoto, Fernando Nestor Facio Júnior, Denise Cristina Móz Vaz Oliani, Antônio Hélio Oliani, Izaura dos Santos Ramos, Júlio César André, Gerardo Maria de Araújo Filho

**Affiliations:** 1 Center for Studies and Development of Health Education—CEDES, São José do Rio Preto Medical School—FAMERP, Avenida Brigadeiro Faria Lima, 5416, Sao Jose do Rio Preto 15090-000, Brazil; daniele.aleixo@edu.famerp.br (D.N.L.A.); pedro.cagnazzo@edu.famerp.br (P.C.C.); leonila.sasso@edu.famerp.br (L.S.d.A.S.); matheus.querino@edu.famerp.br (M.Q.d.S.); emerson.santos@edu.famerp.br (E.R.d.S.); gabriela_longo@yahoo.com.br (G.d.S.L.C.); ana.engel@edu.famerp.br (A.M.R.P.V.T.d.C.E.); janaina.floriano@edu.famerp.br (J.A.d.S.F.); anaflvalencio@hotmail.com (A.B.F.V.); clarissa.nunes@edu.famerp.br (C.A.V.N.); jose.francisco@edu.famerp.br (J.L.E.F.); gustavo.macedo@edu.famerp.br (G.G.M.); dr.andressaks@gmail.com (A.K.S.); bortolucidelima@gmail.com (C.B.d.L.); maysa@famerp.br (M.A.B.); marli@famerp.br (M.d.C.J.); luis.spessoto@famerp.br (L.C.F.S.); fernando.nestor@famerp.br (F.N.F.J.); olianidenise@outlook.com (D.C.M.V.O.); olianiantoniohelio@outlook.com (A.H.O.); izaura.ramos@edu.famerp.br (I.d.S.R.); julio.andre@edu.famerp.br (J.C.A.); gerardo.filho@famerp.br (G.M.d.A.F.); 2 RISE Behaviour Services (Reaching Independence Through Support and Education), Victoria, BC V9A 4J2, Canada; danielleramos@risebehaviourservices.ca; 3 CEDIN—Neurodevelopmental Diagnosis and Intervention Center, Rua Reinaldino Schaffenberg de Quadros, 1485, Alto da XV, Curitiba 80050-435, Brazil; amanda@terapiacedin.com.br; 4 UNIFUNEC—University Center of Santa Fé do Sul, Av. Mangará, 477, Santa Fe do Sul 15775-000, Brazil; 5 UNORTE—University Center of Northern São Paulo, Rua Ipiranga, 3460, Jardim Alto Rio Preto, Sao Jose do Rio Preto 15020-040, Brazil; paulabernardes@unorte.edu.br; 6 UNIVESP—Virtual University of the State of São Paulo, Av. Paulista, 352, 14° Andar, Sao Paulo 01310-905, Brazil; adriana.viviane@yahoo.com.br; 7 FAETI—Iracema College of Education and Technology, Rua Cordeiro da Silva, 165, Sao Paulo 02883-090, Brazil; 8 UNIFEV—University Center of Votuporanga, Av. Nasser Marão, 3069, Pq. Industrial I, Votuporanga 15503-005, Brazil; atr.reismauricio@gmail.com; 9 Graduate Program in Gerontology, UFSCar— Federal University of São Carlos, Rodovia Washington Luís, km 235 (SP-310), Sao Carlos 13565-905, Brazil; forlandi@ufscar.br; 10 University Hospital Center Cova da Beira, University of Beira Interior, 6200-251 Covilha, Portugal

**Keywords:** applied behavior analysis, compassion, interpersonal skills, scoping review, Behavioral Skills Training

## Abstract

Applied Behavior Analysis (ABA) stands at an inflection point, expanding its robust technical framework to embrace the growing demand for humanization and relational excellence. This study, a scoping review, investigated the emergent integration of compassion and related relational/interpersonal skills into ABA practice, justifying its methodology by the need to map a multifaceted and rapidly evolving field. By analyzing 13 articles published between 2020 and 2024, the research revealed a notable acceleration in interest in the topic (76.9% of publications between 2022 and 2024), primarily concentrated in the USA. It was identified that, while the importance of these “soft skills” is valued, there is a gap in formal training. However, Behavioral Skills Training (BST) emerged as an effective and scientifically validated tool for teaching and measuring compassion and relational skills, with positive impacts on both professionals and caregivers. Conceptual articles paved the way for their operationalization. It was concluded that compassion and interpersonal skills are crucial pillars for the evolution of ABA, promoting more effective, ethical, and person-centered interventions. This review not only synthesized current knowledge but also highlighted generalization challenges and directed future research toward an even deeper and culturally relevant impact.

## 1. Introduction

Applied Behavior Analysis (ABA) has established itself as a powerful intervention science, widely recognized for its empirical foundation and effectiveness in modifying socially significant behaviors (Ampuero et al., 2025; Graber & Graber, 2025; Miltenberger et al., 2025; Moore et al., 2026; Silva et al., 2024). Traditionally rooted in radical behaviorism and experimental behavior analysis, ABA has distinguished itself through its data-driven approach, its focus on direct observation, and its capacity to develop precise interventions for a diverse range of contexts, notably in the treatment of Autism Spectrum Disorder (ASD), education, and mental health (Borja et al., 2025; Kalra et al., 2025; McComas et al., 2025). However, the maturity of any scientific field is accompanied by a continuous process of reflection and refinement.

**Figure 1:**
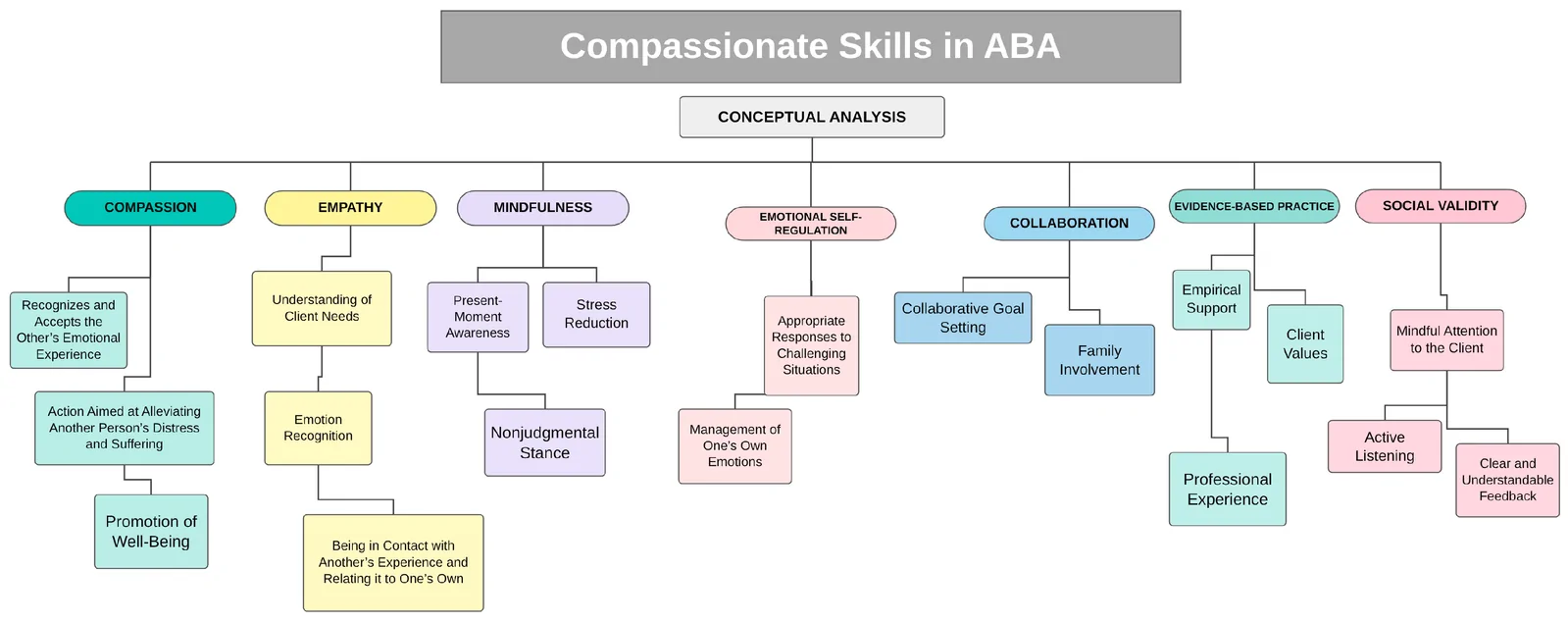
Compassionate Skills in ABA.

In recent decades, this maturation process in ABA has been marked by increasing discussions on aspects that transcend pure behavioral modification (Bonvicini et al., 2009; Kalra et al., 2025; Kristiansen et al., 2025; Malenfant et al., 2022). Issues such as the social validity of interventions (i.e., how acceptable and relevant they are to individuals and the community), the subjective experience of the client and their caregivers, the promotion of a robust therapeutic relationship, and the professional sustainability and well-being of behavior analysts themselves have gained prominence (Beaumont et al., 2016; Cowan & Kodak, 2024; Foxx, 1996; Kranak et al., 2024). This expansion of focus does not represent a deviation from behavioral principles but rather an enhancement and contextualization of their application, aiming to ensure that interventions are not only effective but also humanized, ethically consistent, and culturally sensitive. In this scenario, interpersonal skills, sensitivity, and compassion on the part of ABA professionals have emerged as topics of central interest (Beaumont et al., 2016; Borja et al., 2025; Kalra et al., 2025; LeBlanc et al., 2020; McComas et al., 2025). The demand for approaches that integrate technical excellence with empathetic communication, compassionate management of interactions, and the promotion of effective therapeutic relationships has become pressing, recognizing that treatment adherence, stakeholder satisfaction, and the prevention of professional burnout are intrinsically linked to the quality of human interactions (Figure 1). While previous reviews have explored tangential aspects of this topic, none have investigated the extent and nature of the literature on compassion and interpersonal skills in ABA with the depth and specific focus that this review aims to provide, especially considering the rapid advancements in the field in recent years.

This emphasis is consistent with the very foundations of the discipline. In its classic definition, the term *applied* signals ABA’s commitment to effecting improvements in behaviors that enhance and improve people’s lives, that is, behaviors that are socially significant for the client (Cooper et al., 2020, p. 16). Likewise, the ethical and professional responsibilities of behavior analysts, including their responsibility to clients (Bailey & Burch, 2022; Cooper et al., 2020), as well as interpersonal communication and coping with work-related stress (Bailey & Burch, 2010), have long been framed as core professional repertoires. Taylor et al. (2019) provided an early behavior-analytic account of compassionate care, proposing that treatment outcomes may be enhanced by attending to relationships with caregivers, and more recent contributions have described compassion as an additional dimension of ABA (Penney et al., 2026), discussed how compassion can be scaled and applied in ABA services (Werntz et al., 2026), proposed a multilevel framework to cultivate nurturing systems of compassionate care (Denegri et al., 2026), and explored interprofessional and self-compassion competencies among ABA professionals (Friedman & El-Roy, 2025). Notably, this concern is not exclusive to behavior analysis. In medicine, ‘bedside manner’ has been revisited as a core professional behavior of physicians (Farooq et al., 2013; R. Patel & Wyse, 2025, 2026), and curricula for empathy and compassion training in medical education have been systematically reviewed (S. Patel et al., 2019); in mental healthcare, compassion has been reaffirmed as a basic element of care (Sengupta & Saxena, 2024), and self-care and compassion practices have been incorporated into the training of psychological therapists (Boellinghaus et al., 2013).

Given the emergence and complexity of this theme in the ABA literature, characterized by a growing yet heterogeneous body of publications, a systematic mapping of existing knowledge becomes essential. The choice of a scoping review is fully justified in this context. Unlike systematic reviews or meta-analyses, which aim to answer a highly specific and restricted research question, a scoping review is the most suitable methodology for exploring the extent, nature, and characteristics of the literature on a broad and emerging topic. It allows for the identification of key concepts, the mapping of evidence from different types of studies (from conceptual analyses to empirical trials of various designs), the elucidation of research gaps, and the consolidation of a panoramic view that would otherwise be fragmented. In an area where the definitions of “compassion” and “interpersonal skills” are still being refined within a behavioral framework and where diverse methodological approaches are employed, a scoping review is the ideal tool to synthesize knowledge and guide future investigations.

Thus, the objectives of this study were:

- To identify and characterize published scientific articles addressing compassion and interpersonal skills in Applied Behavior Analysis.
- To describe the methodological designs, studied populations, and main thematic foci of these articles.
- To synthesize the most relevant findings, encompassing theoretical and empirical contributions to the integration of compassion and interpersonal skills into ABA practice.

## 2. Materials and Methods

### 2.1. Methods

This scoping review was conducted to map the scientific literature on compassion and related relational/interpersonal competencies in Applied Behavior Analysis (ABA). The methodology rigorously followed the methodological guidelines established by Arksey and O’Malley (2005) and their practical refinements proposed by Levac et al. (2010). Additionally, recommendations from the Joanna Briggs Institute (JBI) for scoping reviews, detailed in Chapter 10 of the JBI Manual for Evidence Synthesis (Peters et al., 2020), were incorporated to ensure the comprehensiveness and rigor of the process at all methodological stages. Transparency and quality of reporting methods and findings were ensured by adhering to the PRISMA Extension for Scoping Reviews (PRISMA-ScR) guidelines by Tricco et al. (2018). The protocol that guided the performance of this review was published in JMIR and registered in the OSF Open Science Framework.

### 2.2. Research Question

The research question was developed based on the Population, Concept, and Context (PCC) framework, aiming for a comprehensive and delimited exploration of the topic (Page et al., 2021; Peters et al., 2015, 2020, 2024; Tricco et al., 2018). The central research question that guided this study was:

“What has been broadly published in the scientific literature regarding the compassion and related relational/interpersonal competencies of behavior analysts and other ABA practitioners?”

The components of the PCC framework were specifically defined as:

- **Population (P):** Applied Behavior Analysts, understood broadly as individuals who deliver, supervise, or are being trained to deliver ABA services, including certified behavior analysts (e.g., BCBA, BCBA-D, BCaBA), registered behavior technicians (RBTs), clinical supervisors, graduate students/trainees in ABA programs, and other professionals implementing behavior-analytic services (e.g., early intervention providers using behavior-analytic caregiver coaching).
- **Concept (C):** Compassion and related relational/interpersonal competencies. Compassion was treated as the core construct and, consistent with the search strategy, was operationalized to include compassionate care, empathy/empathic responding, caring, the therapeutic relationship, person-centered and humanistic approaches, and the broader set of interpersonal (“soft”) skills through which compassion is enacted in practice (e.g., collaboration with caregivers and other professionals, communication, and self-compassion).
- **Context (C):** Professional practice, training, and supervision in ABA, that is, the settings and processes in which ABA services are delivered (e.g., clinical, home, school, community, and telehealth services) or in which ABA practitioners are trained and supervised (e.g., graduate programs, supervision, and continuing education). Thus, whereas the Concept refers to *what* was examined (compassion and related relational competencies), the Context refers to *where* and *through which professional processes* these competencies are enacted, taught, or evaluated.

To avoid overlap between components, the Population defines who was studied, the Concept defines which repertoires were examined, and the Context defines the professional circumstances in which they occur. Studies were therefore eligible when (a) at least part of the sample consisted of ABA practitioners as defined above (or, for conceptual analyses, when the article addressed this population); (b) the study examined compassion or a related relational/interpersonal competency as defined in the Concept; and (c) these competencies were examined within ABA service delivery, training, or supervision. These operational definitions clarify, rather than modify, the scope of the published protocol, and are directly reflected in the three blocks of the search strategy (Section 2.4; Table A2). No changes were made to the searches, screening, or set of included studies.

### 2.3. Eligibility Criteria

Inclusion and exclusion criteria were formulated to ensure that only relevant studies aligned with the research question were considered, allowing for the capture of the methodological and temporal diversity of the field, as presented in Table A1, in the appendices.

**Participant-related decision rules.** The following rules were applied: (i) studies with students or trainees were eligible when participants were enrolled in ABA training programs or pursuing behavior-analytic certification; (ii) studies with multidisciplinary samples were eligible when ABA practitioners were part of the sample and the competencies examined referred to the delivery of behavior-analytic services (e.g., early intervention providers using behavior-analytic caregiver coaching), in which case data were extracted for the study as a whole and the composition of the sample was recorded (Table S2); (iii) caregivers were not considered a target population, and studies including caregivers were eligible only when caregivers participated as informants about, or recipients of, the compassionate/relational behavior of ABA practitioners (e.g., rating practitioners’ compassionate care or taking part in practitioner–caregiver interactions), or when they contributed to the development of instruments intended for use by ABA practitioners; and (iv) studies in which ABA practitioners were neither part of the sample nor the focus of the article were excluded.

**Definition of conceptual analyses.** Although editorials, expert opinions, and narrative reviews were excluded (Table A1), peer-reviewed conceptual analyses were eligible. For the purposes of this review, a conceptual analysis was defined as a peer-reviewed article that systematically examines a construct within a behavior-analytic framework by (a) explicitly defining and/or operationalizing compassion or related relational competencies in behavioral terms, (b) grounding this analysis in the cited conceptual and empirical literature, and (c) deriving a structured framework, model, or set of behavioral recommendations for ABA practice or training. By contrast, editorials, commentaries, and opinion pieces (i.e., texts primarily expressing the authors’ viewpoints without a structured behavioral analysis of the construct) and narrative reviews (i.e., summaries of the literature without such conceptual operationalization) were excluded. Using this definition, three articles were classified as conceptual analyses (Fiebig et al., 2020; Melton et al., 2023; Rohrer et al., 2021): the first proposes a values-based, three-component model of self-care for behavior analysts; the second analyzes empathy and compassion from a radical behaviorist perspective, differentiating them functionally; and the third analyzes compassionate approaches and “soft skills” as behavioral repertoires amenable to training. These articles were retained only to inform the conceptual mapping of the field (e.g., how compassion is defined and framed) and were not used as evidence of intervention effectiveness; all statements regarding the effectiveness of Behavioral Skills Training (BST) in this review are based exclusively on the included empirical studies (Section 4.2).

*Justifications for Temporal and Access Criteria:*

The period of 2020–2024 was chosen due to the rapid evolution of the field, the paradigm shift towards more person-centered approaches, the impact of the COVID-19 pandemic, recent methodological advances, and updates to policies and guidelines in the area, ensuring the relevance and currency of the findings.

The inclusion of only studies with freely accessible full text, while potentially limiting the scope of the review by excluding relevant studies behind paywalls, prioritized the in-depth analysis of the full content of the selected studies. To mitigate this limitation, an exhaustive search was conducted in multiple databases, and in cases of potentially crucial studies not freely available, direct contact with authors was attempted to request access.

### 2.4. Search Strategy

The search strategy was meticulously developed to be comprehensive and systematic, aiming to identify the greatest possible number of relevant studies. Electronic databases consulted included: Embase, Index Psicologia, Lilacs, PubMed, Scopus, and Web of Science. The selection of these databases was based on their broad coverage of the biomedical, psychological, and health sciences literature, as well as their use in previous systematic reviews in the field of ABA and compassion in healthcare. The search strategies were developed and tested by the research team with the support of a specialized librarian.

Search terms were formulated from combinations of controlled descriptors (MeSH—Medical Subject Headings and DeCS—Health Sciences Descriptors) and free terms (synonyms), in Portuguese, Spanish, and English, according to the PCC mnemonic. Boolean operators “AND” and “OR” were used to refine and expand the search (Table A2 and Table A3—Appendix A).

The search strategy was structured in three blocks that mirror the PCC components (Table A2): (1) a Population block, with terms identifying ABA and behavioral intervention/therapy; (2) a Concept block, with compassion and related relational terms (compassion, compassionate care, empathy, empathic, caring, therapeutic relationship, person-centered, and humanistic approach); and (3) a Context block, with terms related to professional skills and their development (skills, competencies, training, professional development, and therapeutic skills). Related relational competencies that were not used as search terms (e.g., collaboration, interpersonal skills, and self-compassion) were identified in the included studies during full-text review and data extraction when they co-occurred with the Concept terms; their absence as explicit search terms is acknowledged as a limitation (Section 4.4).

The search strategies were adapted according to the specificities of each database, respecting their particular syntaxes and indexing features. All searches were conducted between December 2024 and January 2025, and the results were exported in a format compatible with the reference management software Rayyan (web-based platform; Qatar Computing Research Institute, Doha, Qatar) for subsequent screening and selection.

### 2.5. Study Selection

The study selection process was independently performed by two reviewers (DA and DS) and designed to ensure rigor and minimize bias, according to JBI guidelines. Rayyan software (Ouzzani et al., 2016) was used for reference management and to facilitate study screening.

- **Duplicate Removal:** Initially, all results from database searches were imported into Rayyan, where duplicates were automatically identified and removed by the software and through manual verification.
- **Reviewer Calibration:** Before the actual screening began, reviewers underwent training on the application of inclusion and exclusion criteria. A calibration process was conducted with independent analysis of titles and abstracts of 18 articles. A minimum agreement of 80% between reviewers was established as an acceptance criterion. If the agreement level was not reached, a discussion about discrepancies occurred, criteria were refined, and the calibration process was repeated with a new set of 18 articles until 80% consensus was achieved.
- **Title and Abstract Screening:** In the first phase, the two reviewers independently evaluated the titles and abstracts of the remaining studies against the predefined inclusion and exclusion criteria, classifying each study as “include,” “exclude,” or “uncertain.”
- **Full-Text Review:** Articles classified as “include” or “uncertain” in the previous phase had their full texts retrieved and were independently reviewed by the same two reviewers, applying the detailed eligibility criteria.

Any discrepancies between the reviewers in both phases (title/abstract and full text) were resolved through discussion. In the absence of consensus, a third reviewer (JA) was consulted to mediate and make the final decision. Inter-rater reliability was calculated using Cohen’s Kappa coefficient for both screening phases, with a κ value of 0.8 or higher considered acceptable. A detailed record of all decisions, including reasons for full-text exclusion, was maintained for the construction of the PRISMA flow diagram (Preferred Reporting Items for Systematic Reviews and Meta-Analyses), illustrating the flow of studies throughout the selection stages. As a final quality control measure, a random sample of 10% of the included studies was reviewed by a third assessor (SC) to ensure consistency in the application of inclusion and exclusion criteria.

### 2.6. Data Extraction

Data extraction from the included studies was independently performed by two reviewers (DA and DS), using a standardized and predefined form, according to recommendations for scoping reviews. This form was tested on a pilot sample of five studies and refined as necessary before full implementation, ensuring consistent data collection.

The data extraction instrument was comprehensive, designed to systematically collect relevant information from each study, covering the following aspects (Table A4):

- Study characteristics (e.g., authors, year of publication, country of origin, methodological design).
- Specific ABA information (e.g., intervention description, population served).
- Compassion-related data (e.g., definitions of compassion, specific skills, techniques used).
- Outcomes and results related to compassion.
- Emergent themes and authors’ conclusions.

Any discrepancies during data extraction were resolved through discussion between the two reviewers, with consultation of a third reviewer (JA) when consensus was not reached. The extracted data were compiled and organized in an electronic spreadsheet to facilitate thematic analysis and subsequent synthesis, ensuring traceability and integrity.

### 2.7. Data Synthesis and Analysis

The synthesis and analysis of the extracted data were conducted using a thematic analysis approach, following the six phases outlined by Braun and Clarke (2006). This systematic approach allowed for the identification, analysis, and reporting of patterns (themes) within the data. The process involved:

- **Familiarization with the Data:** Two reviewers (DA and DS) independently read and reread the extracted data, recording initial ideas and observations.
- **Initial Code Generation:** MAXQDA 2024 software (VERBI Software, Berlin, Germany) was used to systematically code relevant characteristics across the entire dataset. A combination of deductive coding (based on research questions) and inductive coding (allowing new themes to emerge directly from the data) was employed.
- **Theme Search:** Codes were grouped and organized into potential themes, gathering all relevant data for each.
- **Theme Review:** Themes were reviewed at two levels: in relation to the coded excerpts (Level 1) and in relation to the complete dataset (Level 2), which allowed for the development of a thematic “map” of the analysis.
- **Theme Definition and Naming:** Continuous analysis was performed to refine the specificity of each theme and the overall narrative of the analysis, culminating in the generation of clear and representative definitions and names for each theme.
- **Report Production:** The final analysis and selection of the most relevant and convincing example excerpts were performed, connecting the findings to the research questions and the existing literature.

To ensure the rigor and transparency of the analysis, the following strategies were implemented: use of a codebook to maintain consistency in coding; investigator triangulation, with two reviewers independently coding and comparing their analyses; and maintenance of a detailed audit trail of analytical decisions. Additionally, member checking was planned, where preliminary findings were shared with a subset of authors from the included studies for feedback.

The types of information that were the primary focus during the analysis included: conceptualizations of compassion within ABA; compassionate skills or behaviors reported in ABA practice; impacts of compassionate approaches on ABA outcomes; barriers and facilitators to implementing compassionate care in ABA; and emergent trends or gaps in the literature regarding compassion in ABA. The presentation of results included both narrative synthesis and visual representations, such as tables and conceptual maps, to offer a comprehensive overview of the current state of knowledge, identify gaps, and suggest directions for future research. This approach allowed for the identification, for example, of the robust methodological diversity of the studies, the predominant behavioral techniques and theoretical frameworks (e.g., BST and ACT), the target skills for training (e.g., interpersonal, self-care, humanized), and the impacts of the intervention (e.g., skill acquisition, professional well-being, social validity), demonstrating the adequacy of the methodology to capture the expected descriptive and analytical findings.

### 2.8. Ethical Considerations

As this was a scoping review of the existing literature that did not involve the direct participation of human beings, formal ethical approval from a research ethics committee was not required. However, all ethical principles of research integrity were rigorously followed, including ensuring accurate, complete, and transparent reporting of methods and results, in addition to due acknowledgment of consulted sources.

## 3. Results

The article selection process across the 6 databases resulted in 86 articles distributed among them. Of these, 5 were removed due to duplication, and 67 articles were deemed ineligible for not fully addressing the guiding question. Subsequently, a total of 14 articles proceeded to abstract reading. At this stage, 1 article was excluded for not meeting the established PCC criteria, after which a total of 13 articles were included in this review (Supplementary Material).

**Figure 2:**
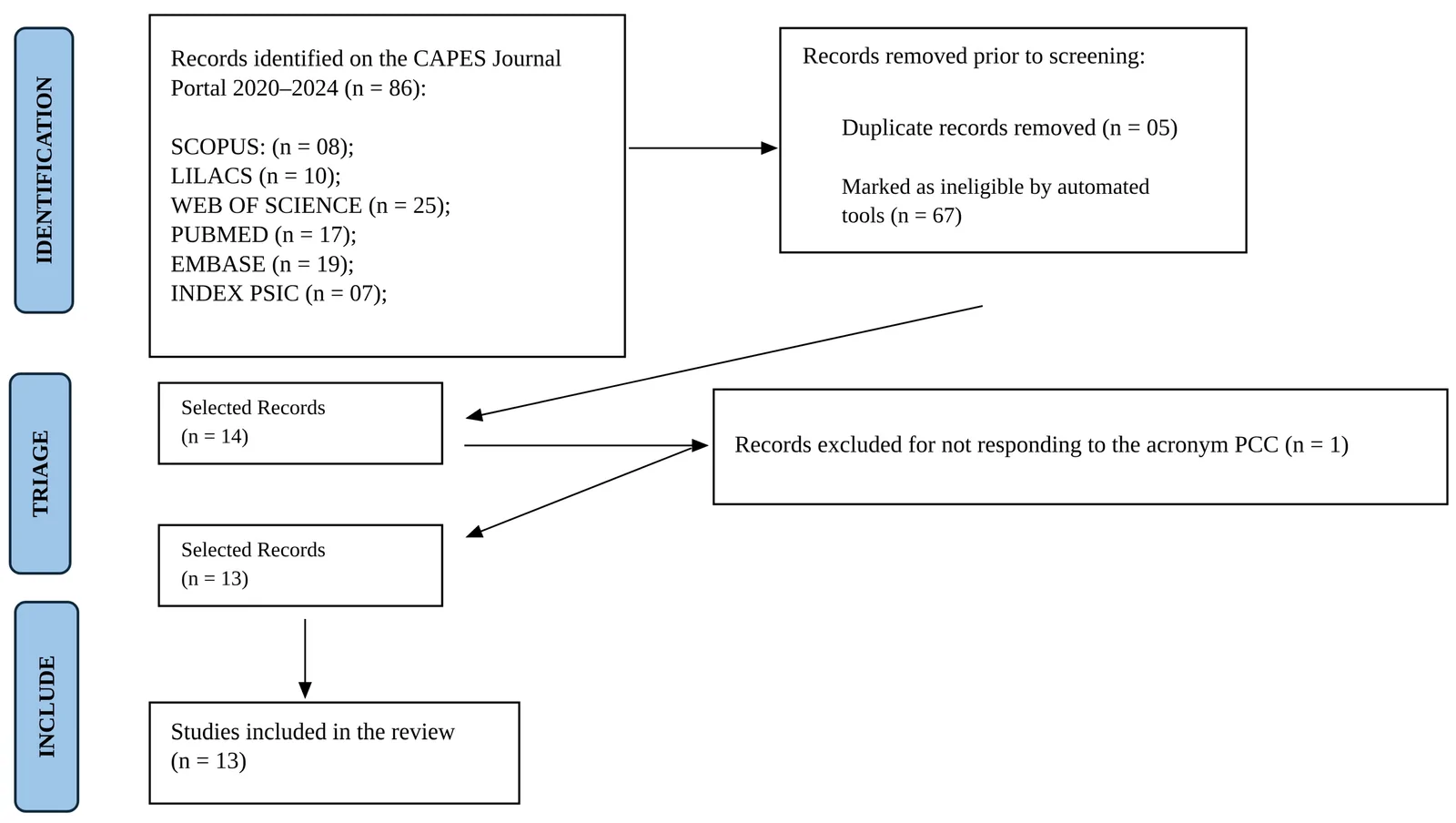
Article selection flowchart.

The present analysis encompassed a total of 13 scientific articles, extracted from the provided material, published between 2020 and 2024 (D’Agostino et al., 2023; Fiebig et al., 2020; Friedman et al., 2024; Gatzunis et al., 2023; Hess et al., 2022; Marchese & Weiss, 2023; Melton et al., 2023; Nohelty et al., 2024; Plattner & Anderson, 2023; Rohrer et al., 2021; Rohrer & Weiss, 2023; Verojporn & Luna, 2024; Zayac et al., 2021)—Figure 2. This selection reflects a notable and growing attention to the integration of compassion skills and related areas in Applied Behavior Analysis (ABA). The majority of these works were published in recent years, with 76.9% of the studies emerging between 2022 and 2024, indicating a deepened interest and the formalization of these practices in the field. Geographically, all identified empirical studies were conducted in the United States of America, suggesting a regional concentration in primary research; see Supplementary Material Table S1.

The sample of articles is divided between empirical studies and conceptual analyses. Out of the total, 10 articles (76.9%) are empirical, directly investigating the acquisition, use, perceptions, or evaluation of skills. The remaining 3 articles (23.1%) are conceptual in nature (conceptual analyses, as defined in Section 2.3), providing theoretical bases and discussions on compassionate approaches and soft skills, self-care, self-compassion, and the functionality of empathy and compassion in ABA. Regarding methodological designs, diversity is striking, reflecting the breadth of research questions addressed. Empirical studies include a variety of approaches: 4 articles (30.8%) employ Single-Case or Experimental Designs (BST), predominant in ABA. Mixed Methods methodologies were used in 2 studies (15.4%), while Quantitative Survey Research, Quasi-Experimental Design, and Randomized Controlled Pilot Study were applied in one study each (7.7% for each type).

Additionally, one article (7.7%) focused on Tool Development and Evaluation. The total participant population in the 10 empirical studies summed to 636 individuals. This diverse sample primarily included Certified Behavior Analysts (BCBAs, BCBA-Ds, BCaBAs, RBTs, LMFTs) and other professionals in the field (such as Early Intervention Providers), who constituted the majority of direct participants. Graduate students in ABA were also a frequent group, especially in studies focused on skills training. Caregivers participated in various studies, either as informants about perceptions or as an integral part of the evaluated interventions, providing crucial data on the social validity and applicability of compassionate care practices.

The main thematic foci of the included articles covered training and interventions for the development of compassion skills, interprofessional collaboration, and mindful self-compassion, explored in 6 studies. Three studies were dedicated to investigating professionals’ perceptions, qualities, and familiarity with compassionate care and the therapeutic relationship. Furthermore, one study focused on the development and evaluation of a tool for parental engagement and compassionate care, and the 3 conceptual articles discussed the theoretical foundation of compassionate approaches and soft skills, the importance of self-care and the differentiation between empathy and compassion.

## 4. Discussion

### 4.1. The Recognition of Importance and Gaps in Formal Training

The literature consistently underscores the high value placed on compassion and interpersonal skills by various stakeholders. D’Agostino et al. (2023), in a mixed-methods study involving 11 early intervention (EI) providers and 28 caregivers, revealed that both groups perceive compassionate care as important, identifying clear benefits such as improved outcomes, strengthened relationships, and increased satisfaction. EI providers assigned an average importance rating between 4.58 and 4.63 (on a 5-point scale) to the compassionate care categories, while caregivers reported averages between 4.17 and 4.46, indicating a strong consensus on the relevance of these skills. However, the same study identified that the perceived self-efficacy of EI providers regarding these skills varied from 3.89 (“somewhat confident”) to 4.35 (“approaching very confident”) across the four categories, with the lowest self-efficacy specifically reported for the collaboration category (mean of 3.89). This suggests a gap between the recognition of importance and confidence in one’s ability to execute.

This gap in formal training is a widely discussed topic in the literature. The study by Rohrer and Weiss (2023), cited by Verojporn and Luna (2024), as well as by Friedman et al. (2024), highlights that the field of behavior analysis would benefit from additional prioritization, instruction, and practice of compassionate care skills in graduate training. LeBlanc et al. (2020), also cited in Rohrer, indicated that many participants (50%) reported that their instruction in interpersonal skills came from mentored practical experiences, and that the majority (83%) indicated that professional training programs should be developed to address compassion, empathy, and relationship-building skills. This finding underlines the need for systematic development of specific curricula. This need is convergent with earlier calls for explicit training in compassionate care and therapeutic relationship skills (Taylor et al., 2019) and with recent qualitative work exploring interprofessional and self-compassion competencies from the perspective of ABA professionals (Friedman & El-Roy, 2025).

Despite recognition, the practical application of these skills can be inconsistent. D’Agostino et al. (2023) quantified that skills such as “offering actions to alleviate caregiver suffering” were reported as used by EI providers in only 38.09% of sessions, and “asking about obstacles that might impede intervention” in only 22.62% of sessions. In contrast, skills like “making positive comments about the child’s behavior” were reported in 98.80% of sessions, and “asking how the caregiver is doing” in 79.76%. This variability in frequency of use highlights that while some skills are frequently employed, others crucial for compassionate care and collaboration are underutilized.

### 4.2. The Effectiveness of Behavioral Skills Training (BST)

In response to the identified training need, Behavioral Skills Training (BST) emerges as a robust and scientifically validated methodology for developing these complex competencies. Importantly, this conclusion derives from the included empirical studies (e.g., Gatzunis et al., 2023; Nohelty et al., 2024; Rohrer & Weiss, 2023; Verojporn & Luna, 2024), and not from the conceptual analyses. Gatzunis et al. (2023), in a study with seven ABA graduate students, demonstrated the effectiveness of telehealth-mediated BST for teaching three skill sets. Mean baseline performance levels were 38.02% for functional assessment interviewing, 47.56% for cultural responsiveness, and 63.63% for empathic/compassionate care. After training, post-training means reached 87.26%, 89.15%, and 90.84% respectively for each skill set. BST was immediately effective in increasing performance to at least 80% for 6 of 7 participants in cultural responsiveness and 5 of 7 in functional interviewing. High social validity was corroborated by the mean rating of 3.97 (out of 4.0) assigned by participants to the training, which led them to “strongly recommend” the training to other ABA professionals.

These findings are consistently replicated. Verojporn and Luna (2024), in a case study with two ABA master’s students (Mei and Ai), showed that a training package (instructions, video modeling, rehearsal, and feedback) significantly increased scores on compassionate care checklists (CCC) and interpersonal skills (IS). Mei, who showed lower baseline CCC scores, achieved higher levels and mastery criteria after training. Ai, whose baseline CCC mean was 68.8%, saw her performance significantly increase. Notably, Ai’s IS score rose from a baseline mean of 83.2% to 100% after training and during generalization phases. Study reliability was ensured by an average procedural fidelity of 95.8% and high levels of interobserver agreement (88.3–95.91% for CCC and 91–98.8% for IS).

Expanding on this evidence, Friedman et al. (2024) conducted a feasibility study with 24 ABA professionals (12 BCBAs, 1 BCaBA, 11 RBTs), applying a four-month training and coaching program. This quasi-experimental design aimed to investigate whether such an intervention could improve self-perceived competencies in interprofessional collaboration (IPC) and mindful self-compassion (MSC). The results were promising, indicating preliminary effectiveness. Analyses revealed a statistically significant improvement in self-perceived interprofessional collaboration competencies, as measured by the Interprofessional Collaborative Competency Attainment Survey (ICCAS), with *p* < 0.001. The total ICCAS scores increased by an average of 0.55 points per question post-intervention.

Notably, the “Team Functioning” subscale within the ICCAS showed the largest average gain of 1.033 points, indicating a substantial improvement in participants’ perception of their ability to function within teams. While the “Collaboration” subscale demonstrated a smaller increase of 0.26667 points and did not reach statistical significance (*p* = 0.171), five of the six ICCAS subscales showed statistically significant improvement. For mindful self-compassion, measured by the Self-Compassion Scale, Short Form (SCS-SF), a statistically significant difference was also revealed in the post-test, with *p* = 0.004. The SCS-SF scores showed an average increase of 0.30556 points per question. The program proved to be efficient, requiring a total investment of no more than 7 h per participant over the four months, demonstrating its potential for practical implementation within busy professional schedules. This study specifically addressed a noted gap by operationalizing collaboration alongside self-compassion, highlighting the importance of integrating collaborative practice within soft-skill awareness and competency, and providing an implementation procedure to improve these skill sets among ABA professionals.

### 4.3. Challenges in Generalization, Cultural Nuances, and Perception Discrepancies

Despite the effectiveness of training in skill acquisition, generalization to real contexts and populations presents significant challenges. Verojporn and Luna (2024) observed that, during the generalization phase, Mei and Ai failed to ask open-ended questions (Response 7) when updating caregivers on progress, suggesting that the application of specific skills may not automatically generalize to all interaction contexts or different tasks (e.g., interviews versus progress updates). Gatzunis et al. (2023) also reported that, for one of the two participants tested for generalization with real caregivers (Mike), performance in “empathic and compassionate care” decreased to 77.3% after 33.75 days from the last training session, down from 98.8% in the last training session. This indicates that long-term maintenance and generalization may require additional reinforcement strategies or booster sessions.

Cultural nuances are a fundamental consideration. Gatzunis et al. (2023) highlighted the difficulty of defining “correct responses” in training packages without considering the cultural context of the family or clinician. They exemplify this with the ambiguity of terms like “technical jargon” or “offensive expressions,” whose interpretation and impact vary culturally. Verojporn and Luna (2024) corroborated this by mentioning that the frequent use of a child’s name, common in some Western cultures, can be perceived as repetitive and unnatural in others, such as in Thailand, underscoring the need for cultural adaptation. The lead researcher of the study, who was a non-native English speaker and grew up in Asian countries, noted that some responses on the Compassionate Care Checklist (CCC) and Interpersonal Skills (IS) would be unacceptable in her culture.

The complexity of measuring and perceiving these skills is evidenced by discrepancies between reports. D’Agostino et al. (2023) identified low average concordance (below 70% in more than half of the pairs, or 18 out of 28 pairs) between EI providers’ and caregivers’ reports on the frequency of compassionate care skill use, particularly in the “compassion” (mean of 63.64%) and “collaboration” (mean of 57.50%) categories. In general, caregivers reported a higher frequency of use by providers than the providers themselves. This discrepancy suggests that professionals’ self-perception may be overly critical or that there is a lack of clarity regarding what constitutes the manifestation of these skills in real-time. Barriers such as limited time, the context of the COVID-19 pandemic, and the virtual modality of sessions were frequently cited by providers as impediments to the consistent application of skills.

In summary, the analyzed body of research demonstrates that the integration of compassion and interpersonal skills into ABA is an ethical and practical imperative. BST is an effective tool for teaching these skills. However, the field still faces the challenge of optimizing generalization to diverse contexts and populations, culturally adapting interventions, and refining the measurement of these competencies in a more objective and aligned manner between parties, ensuring that the application of ABA is profoundly human, effective, and socially validated.

### 4.4. Limitations of the Review

The present review, while comprehensive in its scope, possesses inherent limitations related to its methodology and the scope of the analyzed literature. The preponderance of empirical studies conducted in the United States of America limits the direct generalization of results to other cultural, socioeconomic, and healthcare systems, as the nuances of compassion and interpersonal interactions can vary significantly across different cultures. The inclusion of single-case studies and pilot studies, while essential for exploring an emerging field, restricts the capacity for robust causal inferences and the generalization of results to broader populations. Furthermore, the reliance on self-report measures in several studies, especially those addressing perceptions of interpersonal skills and compassion, can introduce biases, as observed in the discrepancies between providers’ self-perception and caregivers’ reports on the frequency of skill use.

In addition, four methodological aspects should be acknowledged. First, although compassion was operationalized broadly, some related relational competencies (e.g., collaboration, interpersonal skills, and self-compassion) were not used as explicit search terms, which may have reduced the sensitivity of the search for studies indexed only under these terms. Second, the restriction to freely accessible full texts may have excluded relevant studies, even though contact with the authors of potentially relevant paywalled studies was attempted. Third, three conceptual analyses were included (Fiebig et al., 2020; Melton et al., 2023; Rohrer et al., 2021); although they met the definition adopted in this review (Section 2.3), they were used only for conceptual mapping, and their exclusion would not change the conclusions regarding the effectiveness of training procedures, which rest solely on empirical studies. Fourth, the searches were conducted between December 2024 and January 2025, and the literature on compassion in ABA has expanded considerably since then, including a Special Issue of *Behavior Analysis in Practice* (e.g., Denegri et al., 2026; Penney et al., 2026; Werntz et al., 2026) and new qualitative evidence (Friedman & El-Roy, 2025). Some of these works first appeared online within the review period but were not retrieved by the search strategy; they were incorporated into the Introduction and Discussion to contextualize the findings, but were not part of the formal evidence synthesis. An update of this review is therefore recommended.

### 4.5. Future Perspectives and Emerging Novelties in the Thematic

The current landscape of the literature points to fertile directions for future research and practical advancements.

- **More Robust Designs and Larger Samples:** There is a pressing need for studies with more rigorous designs, such as randomized clinical trials and quasi-experimental studies with larger and diverse samples, to validate the effectiveness of training packages at scale and to evaluate the long-term maintenance and generalization of these skills to a variety of clinical settings and client populations.
- **Cross-Cultural Validation and Contextualization:** Future investigations should focus on the cross-cultural validation of interventions, adapting the definitions, teaching, and measurement of compassion and interpersonal skills for different cultural and linguistic contexts, directly addressing the concerns raised by Gatzunis et al. (2023) and Verojporn and Luna (2024).
- **Measurement Refinement:** The development and validation of direct and objective behavioral measures of compassion and relational skills are crucial to complement self-reports and reduce bias, offering a more precise evaluation of the acquisition and application of these competencies. For example, D’Agostino et al. (2023) suggest that the newly developed Parent Partnership Questionnaire (Marchese & Weiss, 2023) has the potential for use in conjunction with training to measure and improve the soft skills of ABA practitioners.
- **Optimization of Technology Use:** Telehealth will continue to be a powerful tool, and research should explore how to optimize it for training and supervision of interpersonal skills, seeking to maximize the quality of interaction and overcome communication and engagement barriers.
- **Integration of Self-Care and Burnout Prevention:** Research should delve into the interrelationship between self-care, self-compassion of professionals, and the quality of compassionate care offered, developing and testing interventions that promote resilience and prevent burnout in ABA practice.
- **Behaviorally Defined Outcomes of Compassionate Care:** To increase relevance and uptake among behavior analysts, future studies should evaluate the effects of compassionate and relational practices on directly observable dependent variables commonly used in ABA research. For example, studies could examine whether clients who participate in goal-setting require fewer trials to criterion, or whether caregivers implement behavior plans with a higher percentage of treatment fidelity when practitioners briefly check in with them at the beginning of each session.
- **Stakeholder Feedback:** Future research and practice should systematically ask clients and their caregivers to identify areas in which practitioners’ relational and compassionate repertoires can improve, operationalize this feedback into observable target behaviors, and use it to refine training curricula and to evaluate social validity, as exemplified by instruments such as the Parent Partnership Questionnaire (Marchese & Weiss, 2023).

Considering the robustness of this scoping review’s findings, the practical implications for the field of Applied Behavior Analysis are substantial and immediate. For ABA educators and training programs, it becomes imperative to systematically and formally integrate modules dedicated to compassion, empathy, relational skills, and interprofessional collaboration into curricula, leveraging Behavioral Skills Training (BST) as a central and empirically validated pedagogical methodology. This integration can involve simulations, role-playing, and structured feedback, mirroring the successes reported in the literature. Analogous to the dedicated continuing education requirements already established for ethics and supervision, specific modules or continuing education units focused on compassionate care could be incorporated into the training and certification pathway of behavior analysts, systematically exposing prospective professionals to ways of integrating compassion into their daily work. For supervisors, the adoption of BST within their supervisory practices can optimize the development of these “soft skills” among their supervisees, focusing not only on skill acquisition but also on their generalization across diverse contexts and populations, with particular attention to cultural nuances. Finally, for practicing ABA professionals, the continuous integration of self-compassion and self-care strategies into their daily work routine is crucial for mitigating burnout. Simultaneously, the active enhancement of compassionate skills, even those less intuitive (e.g., addressing caregiver distress), can further deepen the social validity of interventions, strengthen the therapeutic relationship, and foster more ethical and person-centered outcomes.

By embracing these directions, ABA can further strengthen its position as an applied science that not only transforms behaviors but also nurtures human relationships, promoting care that is effective, ethical, culturally responsive, and profoundly human.

## 5. Conclusions

The review of the 13 articles analyzed herein solidifies the understanding that the integration of compassion and interpersonal skills represents an essential and rapidly expanding frontier for Applied Behavior Analysis (ABA). Recent research demonstrates an increasing recognition of the importance of these “soft skills” to enhance the effectiveness, social validity, and humanization of behavioral interventions.

It was found that the value placed on these qualities by ABA professionals is widespread, although a gap persists in formal training for their systematic acquisition. However, the literature points to promising avenues: Behavioral Skills Training (BST) emerged as a robust and scientifically validated methodology to operationalize, teach, and measure complex skills such as compassionate care, empathy, and collaboration, including in telehealth formats. These findings indicate that ABA not only recognizes the importance of these skills but also develops effective means to incorporate them into its practice.

The application of these trainings has shown a positive impact on both the capacity building of behavior analysts and supervisors and on caregivers’ perceptions, suggesting an improvement in the quality of interactions and satisfaction. Conceptual works provide the theoretical basis for this evolution, differentiating constructs and emphasizing the need for professional self-care for the sustainability of practice.

In sum, ABA is in a continuous movement of self-construction, seeking to complement its technical excellence with a deepened relational sensitivity. The evidence points to a future where behavioral competence and compassion will walk hand in hand, promoting more effective, ethical, and person-centered interventions. This body of research not only validates this trajectory but also lays the groundwork for future investigations and the continuous development of practices that enrich the lives of clients and professionals.

## Data Availability

No new data were created or analyzed in this study. Data sharing is not applicable to this article.

## Supplementary Materials

The following supporting information can be downloaded at: https://www.mdpi.com/article/10.3390/bs16101950/s1, Table S1: Details of included articles in the review; Table S2: Study Characteristics; Table S3: Specific Information about ABA; Table S4: Compassion-Related Data; Table S5: Results and Analyses; Table S6: Themes and Concepts; Table S7: Conclusions and Implications; Table S8: Quality Assessment; Table S9: Reviewer Notes.

## Appendix A

**Table A1:** Eligibility Criteria.

PCC Component	Inclusion Criteria	Exclusion Criteria
**Population (P)**	• Studies involving applied behavior analysts • Professionals working with ABA • Students in ABA training (graduate students/trainees enrolled in ABA programs or pursuing behavior-analytic certification) • Multidisciplinary samples that include ABA practitioners delivering behavior-analytic services • Caregivers only as informants about, or recipients of, practitioners’ compassionate/relational behavior, or as contributors to instruments intended for ABA practitioners	• Studies not focusing on Applied Behavior Analysis • Professionals from other areas unrelated to ABA • Studies in which ABA practitioners were neither part of the sample nor the focus of the article • Studies with caregivers as the sole target population
**Concept (C)**	• Studies presenting compassion concepts in titles, abstracts, or descriptors • Similar terms related to compassion (e.g., “compassionate skills”) • Compassionate approaches linked to ABA • Related relational/interpersonal competencies (e.g., compassionate care, empathy, caring, therapeutic relationship, person-centered/humanistic approaches, collaboration, interpersonal/soft skills, self-compassion)	• Studies not presenting compassion concepts or similar terms linked to ABA • Studies without a focus on compassionate aspects of practice
**Context (C)**	• Specific skills, techniques, and knowledge in ABA • Interpersonal competencies and therapeutic techniques • General approach to client care • Related terms (e.g., “behavioral skills”) • ABA service delivery (clinical, home, school, community, telehealth), training, and supervision settings	• Studies not addressing professional skills or competencies • Contexts unrelated to clinical or professional ABA practice
**General Study Characteristics**	• Peer-reviewed studies • Published in Portuguese, English, or Spanish • Period: 2020–2024 • Full text freely accessible • Quantitative, qualitative, and mixed-methods studies • Experimental and quasi-experimental studies • Randomized and non-randomized controlled studies • Systematic reviews, meta-analyses, meta-syntheses • Single-Case Experimental Designs (SCEDs) • Books and guidelines in indexed sources • Peer-reviewed conceptual analyses (as defined in Section 2.3)	• Editorials, expert opinions (without a structured conceptual analysis) • Narrative reviews • Studies without available full text • Publications outside the established period • Languages other than Portuguese, English, or Spanish

**Table A2:** Search Strategy.

Database	Search Strategy
PubMed	(((“Applied Behavior Analysis”[MeSH] OR “Behavior Therapy”[MeSH] OR “applied behavior analysis” OR “applied behaviour analysis” OR “ABA” OR “behavioral intervention” OR “behaviour intervention” OR “behavioral therapy” OR “behaviour therapy”) AND (“Empathy”[MeSH] OR “compassion” OR “compassionate care” OR “empathy” OR “empathic” OR “caring” OR “therapeutic relationship” OR “person-centered” OR “humanistic approach”)) AND (“Professional Competence”[MeSH] OR “Clinical Competence”[MeSH] OR “skills” OR “competencies” OR “training” OR “professional development” OR “therapeutic skills”))
Scopus	TITLE-ABS-KEY((“applied behavior analysis” OR “applied behaviour analysis” OR “ABA” OR “behavioral intervention” OR “behaviour intervention” OR “behavioral therapy” OR “behaviour therapy”) AND (“compassion” OR “compassionate care” OR “empathy” OR “empathic” OR “caring” OR “therapeutic relationship” OR “person-centered” OR “humanistic approach”) AND (“skills” OR “competencies” OR “training” OR “professional development” OR “therapeutic skills”))
Web of Science	TS=((“applied behavior analysis” OR “applied behaviour analysis” OR “ABA” OR “behavioral intervention” OR “behaviour intervention” OR “behavioral therapy” OR “behaviour therapy”) AND (“compassion” OR “compassionate care” OR “empathy” OR “empathic” OR “caring” OR “therapeutic relationship” OR “person-centered” OR “humanistic approach”) AND (“skills” OR “competencies” OR “training” OR “professional development” OR “therapeutic skills”))
Embase	(‘applied behavior analysis’/exp OR ‘behavior therapy’/exp OR ‘applied behavior analysis’:ti,ab OR ‘applied behaviour analysis’:ti,ab OR ‘ABA’:ti,ab OR ‘behavioral intervention’:ti,ab OR ‘behaviour intervention’:ti,ab) AND (‘empathy’/exp OR ‘compassion’:ti,ab OR ‘compassionate care’:ti,ab OR ‘empathy’:ti,ab OR ‘empathic’:ti,ab OR ‘caring’:ti,ab OR ‘therapeutic relationship’:ti,ab OR ‘person-centered’:ti,ab) AND (‘professional competence’/exp OR ‘clinical competence’/exp OR ‘skills’:ti,ab OR ‘competencies’:ti,ab OR ‘training’:ti,ab OR ‘professional development’:ti,ab)
LILACS	(tw:(“análise do comportamento aplicada” OR “terapia comportamental” OR “intervenção comportamental” OR “ABA” OR “applied behavior analysis”)) AND (tw:(“compaixão” OR “empatia” OR “cuidado compassivo” OR “relacionamento terapêutico” OR “centrado na pessoa” OR “humanístico”)) AND (tw:(“habilidades” OR “competências” OR “treinamento” OR “desenvolvimento profissional” OR “habilidades terapêuticas”))
Index Psicologia	(“análise do comportamento aplicada” OR “terapia comportamental” OR “intervenção comportamental” OR “ABA”) AND (“compaixão” OR “empatia” OR “cuidado compassivo” OR “relacionamento terapêutico” OR “centrado na pessoa”) AND (“habilidades” OR “competências” OR “treinamento” OR “desenvolvimento profissional”)

**Table A3:** Controlled Descriptors and Free Terms by PCC Component.

PCC Component	MeSH/Emtree/DeCS
Population (P) Behavior Analysts	MeSH: Applied Behavior Analysis; Behavior Therapy; Professional Competence Emtree: applied behavior analysis; behavior therapy; professional competence DeCS: Análise do Comportamento Aplicada; Terapia Comportamental; Competência Profissional
Concept (C) Compassion and related relational competencies	MeSH: Empathy; Professional-Patient Relations; Attitude of Health Personnel Emtree: empathy; patient care; health care quality DeCS: Empatia; Relações Profissional-Paciente; Atitude do Pessoal de Saúde
Context (C) Professional Skills	MeSH: Clinical Competence; Professional Development; Education, Professional Emtree: clinical competence; professional development; professional education DeCS: Competência Clínica; Desenvolvimento Profissional; Educação Profissional

**Table A4:** Data Extraction Tool.

Section	Data to Be Extracted
Study Characteristics	- Authors - Year of publication - Country of origin of the study - Language of the article - Study design and methodology (e.g., qualitative, quantitative, mixed methods) - Sample size and participant characteristics (e.g., age, gender, diagnosis if applicable) - Study setting (e.g., clinical, educational, community, home-based)
ABA-Specific Information	- Specific ABA techniques or interventions used - Target behaviors or skills addressed - Duration and intensity of ABA intervention - Qualifications and training of ABA practitioners involved - Theoretical framework or model of ABA used (if specified)
Compassion-Related Data	- Definition or conceptualization of compassion used in the study - Measures or assessments of compassion (if any) - Compassion-related skills or behaviors described - Training in compassionate care (if any) - Integration of compassion into ABA practice (methods and challenges)
Outcomes	- Primary and Secondary outcomes reported - Measures used to assess outcomes - Key findings related to compassion in ABA practice - Statistical analyses used (if applicable) - Effect sizes or other measures of impact (if reported)
Themes and Concepts	- Main themes or concepts related to compassion in ABA - Barriers or facilitators to implementing compassionate care in ABA - Reported impacts of compassionate approaches on ABA outcomes - Ethical considerations related to compassionate care in ABA
Author Conclusions	- Key conclusions drawn by the authors - Implications for practice or future research suggested - Recommendations for policy or guideline development
Quality Assessment (for descriptive purposes only)	- Study limitations reported by authors - Potential sources of bias identified - Generalizability of findings - Adherence to reporting guidelines (e.g., CONSORT, STROBE, COREQ)
Reviewer Notes	- Any additional observations or interpretations by the reviewer - Questions or uncertainties to be discussed with the research team - Potential links or contradictions with other included studies
