# Supplementary material for: Competence and Care: A Scoping Review Exploring the Incorporation of Compassion and Relational Skills in Applied Behavior Analysis
Source: Behav Sci (Basel). 2026 Oct 20;16(10):1950. doi: 10.3390/bs16101950 (PMC13701841; doi:10.3390/bs16101950)
Supplement: Supplementary file 1 [file behavsci-16-01950-s001.zip › behavsci-4559968-supplementary.pdf]

SUPPLEMENTARY MATERIAL

Table S1. Detail of included articles in the review.

Characteristic	***Category***	***Number (n)***	***Percentage (%)***
Type of Approach	Empirical	10	76.9%
	Conceptual	3	23.1%
Methodological Design (Empirical Studies n=10)	Single-Case/Experimental (e.g., BST, Multiple Probe, AB, Multiple Baseline)	4	30.8%
	Mixed Methods	2	15.4%
	Quantitative Survey	1	7.7%
	Quasi-Experimental	1	7.7%
	Pilot Randomized Controlled	1	7.7%
	Tool Development and Evaluation	1	7.7%
	Conceptual Analysis/Review (Non-Empirical)	3	23.1%
Year of Publication	2020	1	7.7%
	2021	2	15.4%
	2022	1	7.7%
	2023	6	46.2%
	2024	3	23.1%
Country of Origin (All studies)	USA	13	100.0%
Total Participants (Empirical Studies n=10)	Overall Total Participants	636	N/A
Participant Type (Empirical Studies n=10)	Professionals (BCBAs, Supervisors, EI Providers, etc.)	~523*	~82.2%*
	Students (Graduate in ABA)	13	~2.0%*
	Caregivers	100	~15.7%*
Main Thematic Focus	Skills Training and Intervention (BST, CCT, IPC, MSC)	6	46.2%
	Professionals' Perceptions, Qualities, and Familiarity	3	23.1%
	Tool Development and Evaluation	1	7.7%
	Conceptual/Theoretical	3	23.1%

Note: The sum of percentages for participant types may not total 100% due to rounding and potential overlap in some counts. The number of "Professionals" is an estimate based on the sum of different types of professionals explicitly mentioned in empirical articles, excluding caregivers and students to avoid double-counting with separate categories. Year of publication follows the version cited in the reference list of the main manuscript. Conceptual articles: Fiebig et al. (2020), Rohrer et al. (2021), and Melton et al. (2023).

Table S2. Study Characteristics

Authors, Year, Country	Design and Methodology	Sample Size and Participant Characteristics	Study Setting	Funding Source and Conflicts of Interest
Fiebig, J.H., Gould, E.R., Ming, S. et al., 2020, USA	Conceptual Analysis and Literature Review	Not applicable (theoretical article)	Not applicable	Not mentioned in the article
Rohrer, J.L., Marshall, K.B., Suzio, C. et al., 2021, USA	Conceptual Analysis and Literature Review	Not applicable (theoretical article)	Not applicable	Not mentioned in the article
Zayac, R.M., Van Stratton, J.E., Ratkos, T. et al., 2021, USA	Mixed Methods Research (Qualitative and Quantitative)	392 Board Certified Behavior Analysts (BCBAs) and Board Certified Behavior Analyst-Doctorals (BCBA-Ds) in the United States	Not applicable (online study)	Not mentioned in the article
Hess, A., Garcia, Y., Gould, E. R., & Catagnus, R., 2022, USA	Concurrent multiple probe design across participants	6 Board Certified Behavior Analysts (BCBAs) supervising Registered Behavior Technicians (RBTs) and individuals pursuing BCBA certification Sessions conducted via Zoom© videoconferencing software	Not applicable	Not mentioned in the article

Authors, Year, Country	Design and Methodology	Sample Size and Participant Characteristics	Study Setting	Funding Source and Conflicts of Interest
D'Agostino, S. R., Dueñas, A. D., Douglas, S. N., & Meadan, H., 2023, USA	Convergent Mixed Methods Design	11 Early Intervention Providers and 28 Caregivers Served by Them	Sessions Conducted In-Person, Telephonically, or Virtually	Funding Received from the Early On Center for Higher Education; No Conflicts of Interest Declared
Gatzunis, K.S., Weiss, M.J., Rosales, S.A., Fahmie, T.A., Syed, N.Y., 2023, USA	Multiple-baseline design across skill sets	Seven graduate students	Conducted entirely online, utilizing the Zoom videoconferencing platform	No funding / No conflicts of interest
Rohrer, J.L., Weiss, M.J., 2023, USA	Experimental Study / Behavioral Skills Training (BST) Intervention delivered via telehealth	N=4 online Master's-level Applied Behavior Analysis (ABA) students (3 females, 1 male), aged 24-25 years	Varied experience in ABA and family communication (formal vs. informal contexts). No prior exposure to courses in ethics and collaboration. Online, conducted via the Zoom videoconferencing platform	Not explicitly stated in the article. Authors declared no relevant conflicts of interest
Plattner, C., Anderson, C., 2023, USA.	Quantitative survey research	77 certified behavior analysts (BCBAs and BCBA-Ds) recruited via social media platforms (Facebook and LinkedIn)/ All participants worked with autistic children and their families	Online questionnaire administered through the Qualtrics platform.	Does not mention specific funding sources/no conflicts of interest declared.

Authors, Year, Country	Design and Methodology	Sample Size and Participant Characteristics	Study Setting	Funding Source and Conflicts of Interest
Friedman, Z.L.,El Roy, D., Kerwin, E., Tirri, G., Broff, A., 2024, USA.	Quasi-experimental design	24 ABA professionals (12 BCBAs, 1 BCaBA and 11 RBTs) from two American companies	Training and sessions provided virtually	Not mentioned. The authors declared no conflicts of interest
Verojporn, A., Luna, O., 2024, USA	Case study with AB design	Two participants, both master's students in ABA program with prior experience in caregiver interaction and BCBA-D supervision	University library conference room (simulations of caregiver interactions). St. Cloud State University (Student-Mentor Collaboration Grant)	No declared conflicts of interest
Nohelty, K., Novack, M.N., Robinson, R.A., Tevis, C.M., Dixon, D.R., 2024, USA	Pilot randomized controlled study with immediate treatment (IT) and delayed treatment control (DTC) group design	Participants: 9 clinical supervisors (BCBAs or LMFTs). Interactions involved 19 caregivers	Community behavioral health agency. Caregiver collaboration sessions were conducted remotely (Zoom), at the agency, or in the caregiver's home. Training was delivered remotely via Zoom	The authors received research support from the Center for Autism and Related Disorders
Melton, B., O'Connell-Sussman, E., Lord, J., Weiss, M. J.,2023, USA	Conceptual analysis	Not applicable (conceptual/theoretical article)	Not applicable	No conflicts of interest declared
Marchese, N.V., Weiss, M.J., 2023, USA	Study of development and evaluation of a tool (Parental Partnership Questionnaire - PPQ) through a multiphase process	Phase 1 – content construction: 10 caregivers of children with ASD; Phase 2 – content analysis: 32 caregivers of children with ASD; Phase 3 – Reliability and Procedural Integrity: 11 caregivers of children with ASD	Phase 1 interviews: Conducted face-to-face (at treatment centers or residences) or via secure online videoconferencing. Phase 2 online survey: Distributed widely through parent and service provider networks, and autism social media (Facebook). Phase 3 PPQ administration:	No funding was received for manuscript preparation. The authors declare no conflicts of interest

Authors, Year, Country	Design and Methodology	Sample Size and Participant Characteristics	Study Setting	Funding Source and Conflicts of Interest
			Conducted by clinicians at ABA treatment centers in North America	

Table S3. Specific Information about ABA

Study (author, year)	ABA Techniques or Interventions	Target Behaviors or Skills	Duration and Intensity of Intervention	Practitioners' Qualifications and Training	Theoretical Framework or ABA Model
**Fiebig et al., 2020**	Not applicable (theoretical article focused on self-care)	Self-care, self-compassion, resilience, flexibility	Not applicable	Discusses the necessity of formal self-care training for behavior analysts.	Three-component values-based self-care model
**Rohrer et al., 2021**	Discussion on compassionate approaches in ABA	Interpersonal skills, collaboration, compassion	Not applicable	Discusses the necessity of formal training in interpersonal skills.	Applied Behavior Analysis with an emphasis on compassionate approaches
**Zayac et al., 2021**	Not applicable (study focused on practitioner qualities)	35 identified qualities, including being ethical, data-driven, analytical	Not applicable	All participants were certified Board Certified Behavior Analysts (BCBAs) or Board Certified Behavior Analysts-Doctoral (BCBA-Ds)	Applied Behavior Analysis
**Hess et al., 2022**	Behavioral Skills Training (BST) combined with values and committed action training from Acceptance and Commitment Therapy (ACT)	Interpersonal Skills, Collaboration, Planning, and Monitoring in Supervision	Sessions occurred 1-2 days per week; total duration not specified	All participants were Board Certified Behavior Analysts ABA combined with principles of ACT	Applied Behavior Analysis

Study (author, year)	ABA Techniques or Interventions	Target Behaviors or Skills	Duration and Intensity of Intervention	Practitioners' Qualifications and Training	Theoretical Framework or ABA Model
**D’Agostino et al., 2023**	Caregiver coaching in Early Intervention	Compassionate care skills within Early Intervention practice	Not specified; data collected across three consecutive sessions	Early Intervention providers (e.g., speech-language pathologists, occupational therapists, physical therapists, special educators)	Early Intervention emphasizing compassionate care
**Gatzunis et al., 2023**	Behavioral Skills Training (BST)	The study focused on teaching and enhancing three crucial skill sets for ABA practitioners: functional assessment interviewing, empathic and compassionate care, and cultural responsiveness	The total training time ranged from 3.65 to 6.05 hours per participant. The intervention was intensive and individualized	Participants were graduate students in ABA; their initial self-reported qualifications indicated that they lacked significant prior training or coursework in the crucial skills addressed	The study is predicated on the principle that complex skills, including "soft skills," can be effectively taught using Behavioral Skills Training (BST), a proven methodology within ABA for skill acquisition
**Rohrer & Weiss, 2023**	Behavioral Skills Training (BST) components included: instruction, modeling (expert videos), behavioral rehearsal (role-play), and feedback. Applied to teach compassionate care skills within the context of caregiver interviews	11 compassionate/empathic care skills, divided into three clusters: Basic Interviewing Skills, Interest in the Family, and Joining the Family	Training sessions in BST (including role-plays) mean = 24 min. Total training duration (excluding post-training probes) per participant varied from 60 to 90 minutes (mean = 1 hr 13 min). Maintenance probes were conducted 2	Participants were Master's-level ABA students. The trainer (first author) conducted the BST. Video modeling utilized experienced clinicians (doctoral-level BCBAs with expertise in psychology and clinical interviewing)	Grounded in ABA principles, Integrated concepts of empathy and rapport from allied fields (e.g., healthcare, psychology), operationalizing them for application within ABA practice

Study (author, year)	ABA Techniques or Interventions	Target Behaviors or Skills	Duration and Intensity of Intervention	Practitioners' Qualifications and Training	Theoretical Framework or ABA Model
			weeks after the final training session.		
**Plattner & Anderson, 2023**	The article does not introduce new ABA techniques or interventions	Compassionate care skills (e.g., empathy, reflective listening, affirmations)	Not aplicable	Only a small percentage received explicit training	Expansion of ABA practice
**Friedman et al., 2024**	Interprofessional Collaboration (IPC). Experiential teaching and practice of communication, role perception and conflict resolution	Soft-skills competencies, interprofessional collaboration (IPC), mindful self-compassion (MSC) and emotional responsiveness	4-month intervention with total workload of maximum 7 hours over 4 months	Training conducted by PI (occupational therapist and BCBA with dual certification) and co-PI (BCBA-D)	Based on mindful self-compassion (MSC) principles, interprofessional collaboration (IPC) competencies and compassionate care approaches, aiming to integrate these soft-skills into ABA practice
**Verojporn & Luna, 2024**	Training package: graphic feedback, written and vocal skill descriptions with rationale, video modeling, rehearsal and feedback	Compassionate care and interpersonal skills: empathic communication, accessible language, active listening, body posture and use of open-ended questions	Variable	Study participants were master's students in ABA. The lead researcher was a master's student in ABA with 5+ years of experience, under supervision of a BCBA-D with 9 years of experience	ABA with emphasis on compassionate care skills and therapeutic relationship

Study (author, year)	ABA Techniques or Interventions	Target Behaviors or Skills	Duration and Intensity of Intervention	Practitioners' Qualifications and Training	Theoretical Framework or ABA Model
**Nohelty et al., 2024**	Behavioral Skills Training (BST) and Acceptance and Commitment Training (ACTr) integrated into a Compassionate Care Training (CCT) program	Compassionate care strategies and interpersonal skills during clinical supervisors' collaboration with caregivers of autistic children. Five-week program with weekly 1-hour individual training sessions, totaling 5 hours	The trained "practitioners" were clinical supervisors	Training was administered by one of two BCBAs with expertise in compassionate care and caregiver collaboration	The study is grounded in ABA principles, incorporating BST to teach behavioral skills and ACTr to promote psychological flexibility
**Melton et al., 2023**	The article is a conceptual analysis that informs the theoretical basis for teaching and practicing compassionate care skills in ABA; it does not describe a specific intervention technique.	Empathy, Compassion, Compassionate care, Therapeutic relationships	Not applicable	The article discusses the need for explicit training for behavior analysts in compassionate care skills, noting that these skills are often assumed or left to work-related clinical training	Radical Behaviorism; Conceptual analysis framework (Mager, 1997); "Circumstances View" of behavior (Friman, 2021)
**Marchese & Weiss, 2023**	Aims to support collaboration between parents and ABA professionals; addresses parent training in ABA and the integration of compassionate care skills in this context	Assess parents' perceptions of intervention goals, procedures, and outcomes, variables that influence parental engagement and satisfaction with parent training plans in ABA	Interview Duration (Phase 1): averaging 57.9 minutes. PPQ Administration Duration (Phase 3): average of 47.7 minutes. Clinician Training (Phase 3): 30 minutes of behavioral skills training (BST) for PPQ administration	Participating Clinicians (Phase 3): Supervisors (graduate level), BCBAs, RBTs, and one Clinical-Administrative Director (master's level). PPQ Administration Training: Clinicians received 30 minutes of BST training conducted by the study's first author	The theoretical foundation of the study is grounded in the importance of parental engagement, social validity, and compassionate care skills within ABA interventions

Table S4. Compassion-Related Data

Study (author, year)	Definition or Conceptualization of Compassion	Compassion Measures or Assessments	Compassion-Related Skills or Behaviors	Training in Compassionate Care	Integration of Compassion in ABA Practice
**Fiebig et al., 2020**	Self-compassion as part of self-care	Self-compassion as part of self-care	Self-compassion, self-kindness, mindfulness	Advocates for the integration of formal training in ABA programs	Explores the integration of compassionate practices within professional practice
**Rohrer et al., 2021**	Compassion as an essential skills for effective collaboration	Collaboration Tool	Empathy, active listening, effective communication	Proposes the possibility of a self-assessment tool as a treatment guide	Discusses the integration of compassionate practices in behavioral intervention
**Zayac et al., 2021**	Compassion mentioned as one of the 35 qualities	Exemplary Behavior Analyst Checklist (EBAC)	Empathy, client-centeredness, interpersonal skills	Not specified	Discussed as part of the qualities of an exemplary behavior analyst
**Hess et al., 2022**	Self-compassion as part of self-care and professional resilience	Self-Compassion Scale - Short Form (SCS-SF)	Self-compassion, psychological flexibility, values-based action	ACT-based values and committed action training	Discusses the integration of compassionate practices in ABA supervision
**D'Agostino et al., 2023**	Based on the categories of Taylor et al. (2019): positive social interactions, empathy,	Online questionnaires, compassionate care logs,	Positive social interactions, empathy, compassion, collaboration	Not provided as part of the study;	Discussed as integral to caregiver coaching in Early Intervention

Study (author, year)	Definition or Conceptualization of Compassion	Compassion Measures or Assessments	Compassion-Related Skills or Behaviors	Training in Compassionate Care	Integration of Compassion in ABA Practice
	compassion, and collaboration	semi-structured interviews		recommendations made for future training	
**Gatzunis et al., 2023**	Compassion is conceptualized in conjunction with empathy, with both considered crucial interpersonal skills for behavior analysts.	Does not provide a unique and explicit operational definition of "compassion"	Engaging in nodding and minimal expressions; Refraining from interrupting; Delivering empathic statements; Summarizing information received; Requesting feedback; listening, reflecting, and responding	Was an integral part of the Behavioral Skills Training (BST) program implemented in the study.	The integration of compassion into ABA practice as a recognized need and a fundamental objective.
**Rohrer & Weiss, 2023**	Operationalizes an abstract concept (compassion) into specific behaviors that can be taught, observed, and evaluated, serving as an example of skill operationalization within an ABA research context	Compassionate Care Measure (CCM)	Active listening, establishing rapport, expressing acknowledgement and understanding, demonstrating familiarity with the situation, showing positive regard, exhibiting open and neutral body language and facial expressions	Behavioral Skills Training (BST) and Acceptance and Commitment Training (ACT) package delivered via Zoom for teaching compassionate skills	It offers empirical evidence that compassionate care skills can be effectively taught to prospective behavior analysts
**Plattner & Anderson, 2023**	Therapeutic alliance; interpersonal skills; genuine care	Did not utilize a direct and specific measure or assessment of compassion	Empathy, reflective listening, affirmations, rapport, effective communication with parents, management of difficult conversations with parents,	Motivational Interviewing is proposed as a tool that could fill the training gap in compassionate care	Argues that ABA practice must extend beyond these traditional dimensions and incorporate "additional skills" related to interpersonal interaction

Study (author, year)	Definition or Conceptualization of Compassion	Compassion Measures or Assessments	Compassion-Related Skills or Behaviors	Training in Compassionate Care	Integration of Compassion in ABA Practice
			increased parental engagement and adherence	and therapeutic relationship	and therapeutic alliance, which have historically received "minimal attention" in the field
**Friedman et al., 2024**	Compassion is conceptualized as one of the "soft-skills" (interpersonal skills – responsive emotional care; emotional attunement with others; integration of humanity in practice; observable and trainable skill)	Self-Compassion Scale-Short Form (SCS-SF)	Self-compassion; emotional responsiveness; flexible and humanized approach	Utilized video modeling techniques; role-play; discussion and reflection; analysis of practitioner videos	Argue that integration of compassion in ABA practice is necessary and imperative to increase social validity and continued prominence of the field
**Verojporn & Luna, 2024**	Compassion is operationally conceptualized through specific behavioral response classes demonstrated during caregiver interactions. Includes collaborative approaches and effective communication	Compassionate Care Checklist (CCC); Interpersonal Skills Data Collection System (IS)	Use of accessible language; positive/understanding statements, positive gestures (eye contact, leaning, open posture)	Sequential training package	Integrate compassionate and interpersonal skills directly into essential behavior-analytic tasks
**Nohelty et al., 2024**	Compassion is defined as the implementation of empathy in action to alleviate others' suffering, demonstrated through behaviors such as	Compassionate Care Measure (CCM)	Compassionate engagement; response to caregivers' challenges/strong emotions; rapport development, values identification and values-directed	The Compassionate Care Training (CCT) program combines BST and ACTr	To integrate compassionate care directly into collaborative interactions between ABA clinical supervisors and

Study (author, year)	Definition or Conceptualization of Compassion	Compassion Measures or Assessments	Compassion-Related Skills or Behaviors	Training in Compassionate Care	Integration of Compassion in ABA Practice
	active listening, acknowledgment and understanding, and responsiveness to caregivers' needs		actions, recognition of strengths and efforts		caregivers to enhance treatment adherence, satisfaction, and patient outcomes
**Melton et al., 2023**	Compassion is conceptualized as distinct from empathy: empathy involves identifying another's experience and relating it to one's own, while compassion is empathy plus intentional actions to alleviate suffering	The article conceptualizes compassion and discusses the need to develop measures based on this conceptualization, but it does not utilize or apply specific measures.	1. Identification of suffering contingencies; 2. Expression of understanding (verbal); 3. Actions to help alleviate suffering	The study highlights the necessity and potential of conceptual analysis to guide the explicit teaching and supervision of compassionate care skills in behavior analyst training programs	ABA practice is viewed as inherently compassionate. The article advocates for the need to advance the incorporation of compassionate care as a value and skill set in ABA practice
**Marchese & Weiss, 2023**	Skills that include nonverbal and verbal behaviors associated with information gathering, active listening, and offering support and partnership	Parent Partnership Questionnaire (PPQ)	Active listening, reflecting statements, validating concerns, statements that reflect teamwork, acknowledgment of parental mental health impact/stress, checking for variables external to direct treatment, flexibility, brainstorming solutions with the family	The study itself trained clinicians to administer the PPQ, which is a tool designed to support the application of compassionate care in practice	Integration is considered fundamental for successful parent-professional relationships and for the delivery of effective interventions. The PPQ was developed to be a resource that supports this integration

Table S5. Results and Analyses

Study (author, year)	Primary and Secondary Outcomes	Measures Utilized	Key Findings	Statistical Analyses	Effect Sizes or Impact Measures
**Fiebig et al., 2020**	Not applicable (theoretical article)	Not applicable	The importance of self-care for burnout prevention and the enhancement of professional efficacy	Not applicable	Not applicable
**Rohrer et al., 2021**	Not aplicable (theoretical article)	Not applicable	Importance of interpersonal skills for ABA effectiveness	Not applicable	Not applicable
**Zayac et al., 2021**	Identification of the qualities of exemplary behavior analysts	Behavior Analyst Checklist (EBAC), questionnaires (an exemplary behavior analyst checklist developed in this study to identify and assess the qualities of Applied Behavior Analysis professionals)	Top 5 qualities: ethical, data-driven, analytical, client-centered, applied	Descriptive analyses, rankings	Not applicable
**Hess et al., 2022**	Primary: Supervision skill acquisition. Secondary: ACT knowledge, burnout, self-compassion, values-based living	Supervision Relationship Task Analysis (SRTA), SCS-SF, Valuing Questionnaire (VQ), Maslach Burnout Inventory (MBI)	All participants achieved mastery in the SRTA and enhanced their general ACT knowledge	Visual analysis of multiple baseline data	Not applicable (single-case study)

Study (author, year)	Primary and Secondary Outcomes	Measures Utilized	Key Findings	Statistical Analyses	Effect Sizes or Impact Measures
**D'Agostino et al., 2023**	Perceptions and reported use of compassionate care skills	Questionnaires, logs, interviews	Both groups rated compassionate care as important; caregivers reported higher frequency of use than providers	Descriptive analyses, ratings, qualitative thematic analysis	Not applicable (mixed methods study)
**Gatzunis et al., 2023**	Primary: BST was effective in increasing response levels<br>Secondary: Social validity, generalization, and training efficiency	Percentage of correct responses and opportunities; occurrence; interobserver agreement (IOA); procedural integrity (PI); and social validity surveys	Baseline levels (lower levels in functional assessment interviewing, moderate levels for cultural responsiveness, and higher levels for empathic and compassionate care); effects of BST; maintenance; generalization; and social validity	Single-case research design, primarily visual data analysis with calculation of percentage of correct responses, reported with means (M) and ranges	Magnitude of changes in participants' performance levels; substantial and clinically significant increase in skill proficiency, and high social validity
**Rohrer & Weiss, 2023**	Primary: Demonstration of the effectiveness of the training package in increasing compassionate care behaviors in supervisors.<br>Secondary: Support and detail the intervention's impact	Compassionate Care Measure (CCM - modified version); Interobserver Agreement (IOA); Treatment Integrity; Social Validity Questionnaire	Effectiveness of compassionate care training. High Reliability and Treatment Integrity. High Social Validity and Acceptability	A combination of rigorous visual analysis, quantitative single-subject design metrics, and descriptive statistics (means and percentages) was employed.	The authors emphasize that Behavioral Skills Training (BST) was effective in teaching complex skills, a finding that corroborates similar research in other fields.

Study (author, year)	Primary and Secondary Outcomes	Measures Utilized	Key Findings	Statistical Analyses	Effect Sizes or Impact Measures
**Plattner & Anderson, 2023**	Primary: Explore the training experiences and practices of behavior analysts (BCBAs); Secondary: Explore BCBAs' familiarity with Motivational Interviewing (MI)	Motivational Interviewing (MI)	Difficulty with Sensitive Topics with Parents; Gap in Formal Training in Therapeutic Relationship Skills; Impact of Experience on Skills Perception; Low Familiarity and Understanding of Motivational Interviewing (MI)	Descriptive analysis/frequencies and percentages for response description	Does not explicitly report effect sizes or other impact measures
**Friedman et al., 2024**	Focused on improving interprofessional collaborative competencies and mindful self-compassion	Interprofessional Collaborative Competency Attainment Survey (ICCAS) and Self-Compassion Scale, Short-Form (SCS-SF)	Significant Improvement in Interprofessional Collaboration and Mindful Self-Compassion	Paired sample T-test and Cohen's d values	Were presented using Cohen's d, which indicates the magnitude of difference between pre- and post-intervention means
**Verojporn & Luna, 2024**	Primary: Percentage of correct responses on the Compassionate Care Checklist (CCC). Secondary: Percentage of intervals with correct responses in Interpersonal Skills (IS)	Compassionate Care Checklist (CCC) (8 items); Interpersonal Skills Data Collection System (IS) (6 items); Simulated caregiver profiles (cooperative, neutral, non-cooperative); Procedural fidelity and interobserver agreement (IOA); Participant feedback questionnaire (informal)	Training package increased the level of correct responses on CCC and IS for both participants; Generalization across different caregiver profiles	Visual data analysis; No inferential statistics were used	Increase in percentage of demonstrated compassionate care and interpersonal skills by participants

Study (author, year)	Primary and Secondary Outcomes	Measures Utilized	Key Findings	Statistical Analyses	Effect Sizes or Impact Measures
**Nohelty et al., 2024**	Primary: Change in Compassionate Care Measure (CCM) scores. Secondary: Assessment of training feasibility. Compassionate Care Measure (CCM) / Social validity survey (5-point Likert scale)	Training demonstrated feasibility and received support from participating clinicians	Descriptive statistics; Independent t-test; Paired t-test; ANOVA	Significant difference of 0.38 in CCM scores between IT and DTC groups, representing a large magnitude effect (d = 1.31). Mean increase of 0.16 in CCM scores from pre- to post-intervention across all participants	Mean increase of 0.18 in individual CCM items, with notable emphasis on "Normalizing Parents' Feelings" (0.48)
**Melton et al., 2023**	Not applicable (conceptual article).	Not applicable (conceptual article).	The article presents a radical behavioral conceptualization of empathy and compassion, distinguishing them by their critical attributes (contingency identification, expression of understanding, and distinct reinforcement contingencies) and variable attributes. Empathy focuses on understanding and is reinforced by social approval or conversational continuation, whereas compassion focuses on action to alleviate suffering and is reinforced by the actual alleviation of that suffering	Not applicable (conceptual article).	Not applicable (conceptual article).

Study (author, year)	Primary and Secondary Outcomes	Measures Utilized	Key Findings	Statistical Analyses	Effect Sizes or Impact Measures
**Marchese & Weiss, 2023**	Development and evaluation of a tool (PPQ) that supports a collaborative approach to parent training in ABA. Improvement of family satisfaction with ABA interventions and better clinical and therapeutic outcomes	Qualitative Analysis; Online Survey; Inter-rater Reliability; Procedural Integrity and Qualitative Feedback	PPQ Development; Social Validity; Reliability; Procedural Integrity; PPQ Clarity; Feasibility; Themes Frequently Endorsed by Parents	Did not involve complex inferential statistical analyses	Measured reliability and procedural fidelity and potential benefits

Table S6. Themes and Concepts

Study (author, year)	Main Themes	Barriers or Facilitators	Reported Impacts	Ethical Considerations
**Fiebig et al., 2020**	1.The significance of values-driven action in self-care; 2.The impact of context on self-care practices; 3.The role of self-compassion in fostering resilience; 4. The imperative for flexibility and adaptability in professional practice during crises; 5. The integration of personal and professional values within behavioral analysis practice	Barriers: pressure for results, lack of formal training. Facilitators: institutional support, mindfulness practices	Potential enhancement in professional efficacy and burnout prevention	Discussion on the balance between personal care and professional responsibilities
**Rohrer et al., 2021**	1.Importance of compassionate care in ABA; 2. Impact of effective collaboration on treatment outcomes; 3.Barriers to the implementation of compassionate approaches; 4. Strategies for developing interpersonal skills; 5.Necessity of formal training in compassionate care	Barriers: Lack of formal training, excessive focus on technical aspects. Facilitators: institutional support, assessment tools	Potential improvement in the effectiveness of interventions and client satisfaction	Discussion on the balance between scientific objectivity and a compassionate approach
**Zayac et al., 2021**	1. Importance of ethical conduct; 2. Data-driven decision-making; 3. Analytical skills; 4. Client-centered approach; 5. Application of behavioral principles	Not directly specified	Potential impact on training and professional development	Ethics as the most important quality identified
**Hess et al., 2022**	1.Importance of values-based action in supervision; 2. Impact of context on supervision practices; 3. Role of self-compassion in promoting resilience; 4.	Barriers: lack of formal training in interpersonal skills. Facilitators: structured training, feedback	Improvement in supervision skills, potential reduction in burnout risk	Discussion on the balance between personal well-being and

Study (author, year)	Main Themes	Barriers or Facilitators	Reported Impacts	Ethical Considerations
	Need for flexibility and adaptability in professional practice; 5. Integration of personal and professional values in behavioral analytic practice			professional responsibilities
**D'Agostino et al., 2023**	1. Importance of compassionate care; 2. Variability in skill utilization; 3. Need for individualization; 4. Impact of the COVID-19 pandemic	Barriers: time constraints, lack of experience, pandemic impacts. Facilitators: shared experiences, established rapport	Improved outcomes, relationship building, satisfaction	Discussion on the balance between compassionate care and session objectives
**Gatzunis et al., 2023**	1.Need for Interpersonal Skills in ABA; 2.Applicability of Behavioral Skills Training (BST); 3.Telemedicine and Remote Training; 4. Social Validity and Clinical Relevance; 5. Operational Definition of Abstract Concepts	Barriers: Demographic Disparity; Lack of Formal Training; Technical Jargon; Difficulty in Defining and Measuring; Telehealth Limitations; Participant Life Events;<br><br>Facilitators: BST Methodologies; BST Components; Examples and Non-examples; Skill Review; Videoconferencing Platforms; Participant Motivation	Substantial Improvement in Practitioner Skills; Maintenance and Generalization of Skills; High Satisfaction and Perceived Utility; External Validation of Quality; Potential for Improving ABA Service Quality	Institutional Review Board (IRB) Approval; Informed Consent; Confidentiality and Privacy; Professional Responsibility; Transparency
**Rohrer & Weiss, 2023**	1.Compassionate Care in ABA Supervision; 2. Skills Training for Supervisors; 3. Integration of Behavioral Methodologies; 4. Single-Subject Research Design in ABA;	Barriers: Small sample size and limited generalizability; Lack of participant diversity; Absence of supervisee outcome measures;Lack of long-term maintenance	Potential Mitigation of Clinician Burnout; Potential Improvement in Employee Retention	Research Ethics Committee Approval; Informed Consent; Data Confidentiality and Privacy; Voluntariness

Study (author, year)	Main Themes	Barriers or Facilitators	Reported Impacts	Ethical Considerations
	5. Addressing Professional Burnout and Retention	measures and potential for measurement reactivity<br>Facilitators: Evidence-based intervention design; High treatment integrity; High data reliability; High social validity; Relevance to an existing problem		and Protection Against Coercion; Beneficence and Non-Maleficence; Social Validity
**Plattner & Anderson, 2023**	Therapeutic relationship skills with compassionate approach in ABA	Barriers: Nature of self-report (biases); lack of direct behavioral measures; nature of the sample; depth in interview assessment. Facilitators: Addressing a Relevant Research Gap; Contribution to ABA Literature; Methodological Justification for Self-Report; Expert Review; Robust Practical Implications; Significant sample for a survey	Negative impacts related to training gap and BCBAs' skills, and positive potential impacts could be achieved if ABA more effectively incorporated training in therapeutic relationships and Motivational Interviewing	Ethics Committee approval; Informed Consent; Confidentiality, Absence of conflicts of interest
**Friedman et al., 2024**	1.Development of Soft Skills in ABA professionals; 2. Compassion 3. Mindful Self-Compassion	Barriers: Instrument bias; limited sample size; researcher bias; limited professional representation. Facilitators: structured and flexible format; focus on relevant gaps; solid theoretical foundation; distributed practice; leadership expertise	More responsive, compassionate and collaborative practices; development of soft skills in conjunction with hard skills	Ethics Code approval; Informed Consent; Data Privacy and Sensitivity and Conflict of Interest Declaration
**Verojporn & Luna, 2024**	1.Training of compassionate care skills in ABA; 2. importance of interpersonal skills; 3. stimulus and response generalization; 4. addressing gaps in ABA graduate training	Barriers: Measurement limitations; difficulty in generalizing open-ended questions across all contexts; cultural considerations; use of an AB design that limits causal conclusions. Facilitators: Structured training package	Increase in participants' compassionate care and interpersonal skills, replication of previous studies, and extension of	Obtaining signed consent from participants. Study approval by a research ethics committee

Study (author, year)	Main Themes	Barriers or Facilitators	Reported Impacts	Ethical Considerations
		(modeling, rehearsal, feedback); exposure to diverse caregiver profiles	research on skill generalization	
**Nohelty et al., 2024**	1.Need for compassionate care in ABA; 2. Development and evaluation of a compassionate care measure and training program; 3. Feasibility and efficacy of ABA-based interventions (BST and ACTr) for enhancing interpersonal skills;4. The importance of caregiver collaboration in ASD treatment	Barriers: High attrition rate due to time commitment and study logistics (video recording); small sample size; inconsistency in video collection; potential selection bias in supervisors' self-identification of caregivers; excessive focus on new skills may have affected fundamental skills. Facilitators: Overwhelming support from clinicians who completed training; remote training format; the intervention requires minimal additional time in participants' schedules	Improvement in participants' compassionate care scores and reduction in score variance among caregivers for the same supervisor	All procedures approved by ethics committee. Informed consent obtained from all individual participants (supervisors) and written permission for video recording from caregivers
**Melton et al., 2023**	1.Functional differentiation between Empathy and Compassion from a radical behavioral perspective; 2. The central role of reinforcement contingencies in shaping and maintaining these behaviors; 3. Implications of conceptual analysis for the training and supervision of behavior analysts; 4. The inherently compassionate nature of Behavior Analysis and the "Circumstances View"; 5. The need for continuous conceptual and	Barriers: Lack of clear operational definition and explicit training in therapeutic/interpersonal skills. Facilitators: The conceptual analysis framework provides a basis for definition and teaching; inclusion of compassion in the BACB Ethics Code; historical work on social validity and ethics	Potential to guide and refine teaching and supervision, leading to greater technicality and a more compassionate implementation of behavior analysis	Discusses the historical and current relevance of ethical considerations and the new BACB Ethics Code, which emphasizes "treating others with compassion, dignity, and respect"

Study (author, year)	Main Themes	Barriers or Facilitators	Reported Impacts	Ethical Considerations
	experimental advancement in ABA to address complex phenomena			
**Marchese & Weiss, 2023**	1.Development and Validation of Tools; 2. Compassionate Care in ABA; 3. Parental Engagement and Satisfaction; 4. Social Validity; 5. Clinician-Caregiver Relationship	Barriers: Limited tools; Resistance to social validity; COVID-19. Facilitators: The PPQ itself; Semi-structured interviews; Expert and parent feedback; BST training	Provided a resource to support clinicians' work with families; that qualitative analysis can be integrated into ABA research; support for developing positive therapeutic relationships between clinicians and parents	The study implicitly addresses a crucial ethical dimension in ABA practice: the responsibility to listen to and integrate parents' perspectives, promoting more humane and effective care

Table S7. Conclusions and Implications

Study (author, year)	Authors' Key Conclusions	Implications for Future Practice or Research	Recommendations for Policies or Guidelines
**Fiebig et al., 2020**	Self-care is crucial for maintaining efficacy and preventing burnout among behavior analysts	The imperative for research concerning the implementation and efficacy of self-care practices within Applied Behavior Analysis	The inclusion of self-care and self-compassion training within Applied Behavior Analysis educational programs
**Rohrer et al., 2021**	Interpersonal skills are crucial for the growth and effectiveness of ABA	Necessity of research on the implementation and effectiveness of compassionate approaches in ABA. Need to explore validated tools and instruments in ABA	Inclusion of interpersonal skills training in ABA curricula
**Zayac et al., 2021**	Identification of 35 crucial qualities for exemplary behavior analysts	Necessity for research incorporating client and other stakeholder perspectives	Utilization of the EBAC for curriculum and professional development
**Hess et al., 2022**	The incorporation of ACT skills, particularly values and committed action, can enhance supervision relationships in ABA	Necessity for research on the implementation and efficacy of ACT-based practices in ABA supervision	Inclusion of training in interpersonal skills and ACT within ABA training programs
**D'Agostino et al., 2023**	Compassionate care is valued and beneficial; however, its implementation demonstrates variability	Need for continuous training and ongoing support in compassionate care skills	Integration of compassionate care training into Early Intervention provider preparation programs

Study (author, year)	Authors' Key Conclusions	Implications for Future Practice or Research	Recommendations for Policies or Guidelines
**Gatzunis et al., 2023**	Behavioral Skills Training (BST) has been demonstrated as an effective, efficient, and socially valid method for teaching a variety of essential skills to ABA practitioners.	Inclusion of BST in curricula and training programs for developing crucial interpersonal skills; Continuous training Feasibility in telemedicine; Enhancement of the therapeutic relationship;<br><br>Replication studies; Generalization to in-person contexts; Refinement of definitions; Optimal skill Frequency; Group training formats; Long-term maintenance; Exploration of understudied skills	The findings and discussions strongly imply that Regulatory Bodies should consider a more explicit and robust inclusion of training in cultural sensitivity, empathy, and compassion within certification requirements and approved curricula for behavior analysts; that undergraduate ABA training programs should integrate BST as a methodology for teaching interpersonal skills; and that ongoing professional development focused on these skills should be encouraged for currently practicing professionals
**Rohrer & Weiss, 2023**	The evidence-based training package, combining didactic instruction, Behavioral Skills Training (BST), and Acceptance and Commitment Training (ACT), was efficacious in increasing compassionate care behaviors among ABA supervisors	Validated training tool; Enhancement of Supervision Quality; Potential for Reducing Burnout and Improving Staff Retention; Fostering a More Compassionate Work Environment; Generalization to Larger and More Diverse Samples; Measurement of Supervisee (Clinician) Outcomes; Evaluation of Long-Term Skill Maintenance; Component Analysis of the Training Package; Adaptation for Other Contexts and Populations	Development of policies and guidelines that formalize and actively promote compassionate care in ABA supervision, with the aim of fostering the well-being and sustainability of the workforce in the field
**Plattner & Anderson, 2023**	Deficiency in Therapeutic Relationship Skills; ABA professionals with low familiarity in Motivational Interviewing; Need for training in compassionate skills	For practice, comprehensive training; incorporate MI into professional practices/ For research, efficacy of MI training in ABA; Impact on professionals; Behavioral Mechanisms of MI	Curricular review; Training standards; Faculty/Supervisor capacity building; Continuing education

Study (author, year)	Authors' Key Conclusions	Implications for Future Practice or Research	Recommendations for Policies or Guidelines
**Friedman et al., 2024**	A training sequence is a viable and effective procedure for improving "soft skills," specifically Interprofessional Collaboration competencies	Need for soft skills training for professionals in the field; investment in these skills for talent retention. Future research should incorporate direct and observable measures of collaboration and compassion skills; expansion and replication and new assessment tolos	Integration of "Soft Skills" in Professional Training and Development and Promotion of a Collaborative and Empathetic Environment
**Verojporn & Luna, 2024**	The training package was effective in enhancing compassionate care skills among graduate students in ABA during simulated interactions, demonstrating stimulus and response generalization	Practice: Greater prioritization of compassionate skills instruction in ABA graduate training programs. Research: Need for more rigorous studies; recruitment of participants with less experience; investigation of additional measures; inclusion of cultural considerations	ABA should prioritize, instruct, and practice compassionate care skills in graduate training programs
**Nohelty et al., 2024**	The pilot study demonstrates feasibility and overall positive improvement in participants' compassionate care scores following training	Practice: Compassionate care training should be more prioritized in behavior analyst education. Research: Need to collect long-term maintenance data, address selection biases, investigate CCM validity and reliability, individualize training, and evaluate secondary outcomes	Reinforces the principle of the Professional and Ethical Compliance Code for Behavior Analysts (2020) to "treat others with compassion, dignity, and respect," suggesting advancement of this initiative through measures and training programs
**Melton et al., 2023**	Empathy and compassion are distinct yet often interconnected behavioral contingencies. Radical behavioral conceptual analysis provides a framework for their critical and variable attributes, which is fundamental for refining training	Experimentally analyzing the reinforcement contingencies and variable attributes of compassion; expanding research to clients and the broader community; evaluating how skill demonstration is influenced by experience and how critical attributes can be objectively assessed; conceptually analyzing other complex constructs (e.g., self-compassion); integrating critical	Implicitly supports the inclusion of explicit training in compassionate care skills in graduate programs and supervision in ABA, aligning with the ethics code

Study (author, year)	Authors' Key Conclusions	Implications for Future Practice or Research	Recommendations for Policies or Guidelines
	and supervision in compassionate care within ABA	attributes into the training and supervision of behavior analysts	
**Marchese & Weiss, 2023**	The PPQ is a developed and evaluated tool that supports a collaborative approach to parent training in ABA	Clinicians can use the PPQ to collect valuable information about parents' needs and preferences, personalizing parent training plans in ABA. Research: evaluate how clinicians incorporate PPQ responses into treatment plans	Strongly suggests training in compassionate care skills for behavior analysts and the use of tools like the PPQ to facilitate collaboration and social validity in practice

Table S8. Quality Assessment

Study (author, year)	Limitations Reported by Authors	Potential Sources of Bias	Generalizability of Results	Adherence to Reporting Guidelines
**Fiebig et al., 2020**	Not explicitly specified	Potential selection bias in the reviewed literature	Primarily applicable to behavior analysts, but also of potential relevance to other healthcare professionals	Not explicitly mentioned
**Rohrer et al., 2021**	The article provides a practical tool (Compassionate Collaboration Tool); however, it reports on the importance of testing reliability and validity.	Possible self-report bias; necessity for the field to explore and identify means to empirically assess and enhance our own performance in delivering compassionate behavior-analytic treatment.	Not mentioned.	Aligns with existing literature, demonstrating that compassionate skills are important for service delivery (Hojat et al., 2013) and that we are deficient in demonstrating these skills according to consumer research (Taylor et al., 2018).
**Zayac et al., 2021**	Sample limited to BCBAs and BCBA-Ds from the USA	Potential self-selection bias	Results may be specific to the U.S. context	Not explicitly mentioned
**Hess et al., 2022**	Small sample size, lack of in vivo generalization data	Potential selection bias, experimental demand effects	Results may be specific to the U.S. context and ABA supervision	Not explicitly mentioned
**D'Agostino et al., 2023**	Small, homogeneous, and single-state sample; data collection during the pandemic	Potential self-report bias, pandemic effects	Findings may be specific to the U.S. context and Early Intervention	Not explicitly mentioned

Study (author, year)	Limitations Reported by Authors	Potential Sources of Bias	Generalizability of Results	Adherence to Reporting Guidelines
**Gatzunis et al., 2023**	Limitations: The study was conducted during the early stages of the COVID-19 pandemic. Limited practice with non-experimenters. The study was conducted entirely via Zoom, and it remained unclear whether the demonstration of these specific skills would generalize to an in-person interview context. Defining some target skills proved challenging. Frequency of skill demonstration. Learning history of expert evaluators. Difficulty in synthesizing skills. Generalization and maintenance	Potential Biases: Selection bias. Experimenter bias. Response bias (Social Validity). Measurement bias. Attrition bias. Lack of blinding	Generalization to populations (graduate students). Generalization to settings (future research suggested for testing). Generalization of skills (demonstrated generalization to a "real" interview context with parents). Generalization over time (skills were maintained for a period)	Demonstrates strong adherence to reporting guidelines for ABA research, with robust descriptions and clarity of procedures and materials for replication, operationally defined measures, and clearly presented results
**Rohrer & Weiss, 2023**	Limitations: Sample size and diversity; Absence of supervisee outcome measures; Lack of long-term maintenance measures; Potential for measurement reactivity	Participant bias (reactivity to measurement); Selection bias (sample homogeneity); Attribution bias (uncontrolled confounding in small samples)	Limited generalizability to other participants (due to small group size); Limited generalizability to other settings/contexts (as the research was conducted in a specific environment); The study proposes expanding generalization probes to include interactions with actual caregivers and to evaluate the direct impact of this training on clinical outcomes	Strong adherence to ABA research and reporting guidelines and standards, including the implementation of a robust research design, measurement of dependent variables, data reliability, treatment integrity, comprehensive data analysis, social validity considerations, and ethical compliance

Study (author, year)	Limitations Reported by Authors	Potential Sources of Bias	Generalizability of Results	Adherence to Reporting Guidelines
**Plattner & Anderson, 2023**	Dependence on self-report measures	Selection bias; Context bias; Self-report bias	Limited primarily to behavior analysts (BCBAs) working with autistic children and their families	Good adherence to reporting guidelines/Publication in peer-reviewed journal; ethical details; clear methodology; transparent results; declared absence of conflicts of interest
**Friedman et al., 2024**	Self-perception measures; limited sample size; restricted professional representation; insufficient needs assessment	Self-perception tools as possible source of bias; researcher bias; confirmation and sampling bias	Limited generalization	Strong compliance and adherence to scientific research reporting guidelines
**Verojporn & Luna, 2024**	AB design (limits causal inferences); participants already possessed clinical experience; participants' failure to employ open-ended questions during the progress update generalization phase; standardized "correct responses" without cultural context consideration; use of simulations (uncertain whether skills would generalize to actual caregivers)	Selection Bias; Observer/Researcher Bias; Measurement Bias	Limited	Study adheres to standard scientific reporting framework by providing methodological details and transparency regarding limitations
**Nohelty et al., 2024**	Did not collect maintenance data; high attrition rate; supervisors self-identified caregivers (potential selection bias); CCM development is an initial step, requiring validity and	Attrition/Survival Bias; Selection Bias; Observer Bias; Response Bias	Limited	Exemplary adherence to reporting guidelines for a scientific study

Study (author, year)	Limitations Reported by Authors	Potential Sources of Bias	Generalizability of Results	Adherence to Reporting Guidelines
	reliability investigation; training was not individualized (did not address idiosyncratic deficits); focus solely on supervisor performance, not on secondary outcomes for caregivers			
**Melton et al., 2023**	The analysis of self-compassion was beyond the scope; there is a need for more conceptual and practical work on critical attributes; existing research focuses primarily on professional-stakeholder interaction and needs to be extended to clients and the broader community	Not applicable (conceptual analysis)	The proposed conceptual framework is intended to be applicable and informative for understanding and teaching empathy and compassion from a radical behavioral perspective.	Not specified
**Marchese & Weiss, 2023**	Small sample; question influence; formulation bias; no integration guidance; clinician experience; integrity checklist	Selection bias; response bias; observer/administrator bias; sampling bias	Limitation	The article details the methodology of each phase of PPQ development and evaluation

Table S9. Reviewer Notes

Study (author, year)	Additional Observations	Questions or Uncertainties	Potential Connections or Contradictions with Other Studies
**Fiebig et al., 2020**	The article provides practical tools and resources for self-care	How to effectively measure the impact of self-care practices on professional efficacy?	This is consistent with existing research on burnout among healthcare professionals, thereby broadening the scope to include behavior analysts.
**Rohrer et al., 2021**	The article provides a practical tool (Compassionate Collaboration Tool)	How to effectively teach interpersonal and compassionate skills to ABA professionals?	Aligns with studies on the importance of therapeutic and compassionate relationships in other mental health fields
**Zayac et al., 2021**	The study provides a useful tool (EBAC) for assessing professional qualities	How do client perspectives align with those of professionals?	Corroborates studies on the importance of ethics and technical skills in ABA, but also highlights interpersonal qualities
**Hess et al., 2022**	The study provides a practical tool (SRTA) for the assessment of supervision skills	How to effectively implement ACT skills training within ABA programs?	Aligns with existing research emphasizing the importance of self-care and interpersonal skills among mental health professionals
**D'Agostino et al., 2023**	The study yields valuable insights regarding the perception and utilization of compassionate care in Early Intervention	How to effectively operationalize and measure the impact of compassionate care on child and caregiver outcomes?	This aligns with research on the significance of family-centered care in early intervention

Study (author, year)	Additional Observations	Questions or Uncertainties	Potential Connections or Contradictions with Other Studies
**Gatzunis et al., 2023**	This study is of great relevance to the ABA field, addressing a critical gap in professional training: interpersonal skills and cultural sensitivity	How could future research explore more refined methods to capture nuances of subjective terms that pose a challenge for objective measurement?	The study aligns with and reinforces the growing literature pointing to the urgent need for enhanced training in cultural sensitivity and compassion in ABA. No direct contradiction with existing literature was identified in the article
**Rohrer & Weiss, 2023**	Provides a preliminary step, demonstrating that compassionate care skills through BST and ACT training are teachable to ABA students via remote platform and that changes are notable and socially valid in others' perception	Will the compassionate care behaviors acquired by supervisors be maintained for an extended period after training?	Aligns with existing literature, however, has limitations in addressing complex problems such as burnout, which is frequently attributed to broader systemic factors in the literature
**Plattner & Anderson, 2023**	Provides concrete solution proposal; Interdisciplinary Comparison; Identification of challenging topics	How can BCBAs themselves recognize the need for improvement in their therapeutic relationship skills?	Contradictions: Methodology vs. ABA Preference (The study uses self-report, which contradicts ABA's traditional preference for direct observation); ABA professionals' perception of MI/Connections: Confirmation of Skills Deficit; Relevance of Therapeutic Alliance; MI Potential
**Friedman et al., 2024**	The study is situated within a contemporary and important discussion within the ABA field regarding the need to incorporate "soft skills" and more compassionate approaches into practice	How do improvements in professionals' "soft skills" correlate with direct outcomes for individuals served and their families?	The study reinforces and expands the idea that "soft skills" are crucial professional competencies in the ABA context
**Verojporn & Luna, 2024**	Preliminary case study exploring the feasibility of a training package	Are there superior measures for assessing compassion and interpersonal skills in ABA?	Replicates and extends previous studies (Taylor et al., Rohrer et al., Rohrer & Weiss) that called for and/or explored compassionate skills training in ABA, providing preliminary

Study (author, year)	Additional Observations	Questions or Uncertainties	Potential Connections or Contradictions with Other Studies
			evidence of an effective training package and demonstrating generalization
**Nohelty et al., 2024**	The study represents an important step toward formal integration of compassionate care in ABA, which has been criticized for being perceived as "harsh" or "abrasive"	Are the improvements observed in supervisors perceived as compassion by caregivers?	Aligns with and expands previous research highlighting the need for "soft skills" in ABA (Critchfield et al., 2017; LeBlanc et al., 2020) and studies that developed compassion training and tools (Rohrer et al., 2021; Taylor et al., 2019; Canon & Gould, 2022; Rohrer & Weiss, 2022)
**Melton et al., 2023**	The article addresses a gap by offering a conceptual analysis of empathy and compassion from a radical behavioral perspective, a topic that had been largely identified only topographically	The authors question whether merely measuring the behavioral products of compassion and empathy is sufficient, and whether other critical attributes remain to be identified	Connects with Wolf's (1978) work on social validity and Goldiamond's (1974) on ethical implications. Complements previous topographical analyses (Rohrer et al., 2021; LeBlanc et al., 2020; Taylor et al., 2019) by providing a conceptual foundation. Distinguishes empathy and compassion, which are sometimes used synonymously.
**Marchese & Weiss, 2023**	Highlights that qualitative analysis can be a systematic part of behavior-analytic research, achieving high inter-rater agreement	The PPQ integrates social validity measurement at the individual family level. How will clinicians effectively incorporate PPQ responses into treatment plans?	The study connects with and complements existing literature demonstrating the importance of parental involvement in ABA, the relevance of social validity, and the need for compassionate care skills in ABA professionals (and other health professions)
